# Spectral image contrast-based flow digital nanoplasmon-metry for ultrasensitive antibody detection

**DOI:** 10.1186/s12951-021-01188-6

**Published:** 2022-01-04

**Authors:** Sheng-Hann Wang, Chia-Wen Kuo, Shu-Cheng Lo, Wing Kiu Yeung, Ting-Wei Chang, Pei-Kuen Wei

**Affiliations:** 1 Research Center for Applied Sciences, Academia Sinica, Taipei, 11529 Taiwan; 2 Institute of Applied Mechanics, National Taiwan University, Taipei, 11221 Taiwan

**Keywords:** Gold nanoparticles, Local surface plasmon resonance (LSPR), Spectral image contrast, Digital SPR, SARS-CoV-2

## Abstract

**Background:**

Gold nanoparticles (AuNPs) have been widely used in local surface plasmon resonance (LSPR) immunoassays for biomolecule sensing, which is primarily based on two conventional methods: absorption spectra analysis and colorimetry. The low figure of merit (FoM) of the LSPR and high-concentration AuNP requirement restrict their limit of detection (LOD), which is approximately ng to μg mL^−1^ in antibody detection if there is no other signal or analyte amplification. Improvements in sensitivity have been slow in recent for a long time, and pushing the boundary of the current LOD is a great challenge of current LSPR immunoassays in biosensing.

**Results:**

In this work, we developed spectral image contrast-based flow digital nanoplasmon-metry (Flow DiNM) to push the LOD boundary. Comparing the scattering image brightness of AuNPs in two neighboring wavelength bands near the LSPR peak, the peak shift signal is strongly amplified and quickly detected. Introducing digital analysis, the Flow DiNM provides an ultrahigh signal-to-noise ratio and has a lower sample volume requirement. Compared to the conventional analog LSPR immunoassay, Flow DiNM for anti-BSA detection in pure samples has an LOD as low as 1 pg mL^−1^ within only a 15-min detection time and 500 μL sample volume. Antibody assays against spike proteins of SARS-CoV-2 in artificial saliva that contained various proteins were also conducted to validate the detection of Flow DiNM in complicated samples. Flow DiNM shows significant discrimination in detection with an LOD of 10 pg mL^−1^ and a broad dynamic detection range of five orders of magnitude.

**Conclusion:**

Together with the quick readout time and simple operation, this work clearly demonstrated the high sensitivity and selectivity of the developed Flow DiNM in rapid antibody detection. Spectral image contrast and digital analysis further provide a new generation of LSPR immunoassay with AuNPs.

**Graphical Abstract:**

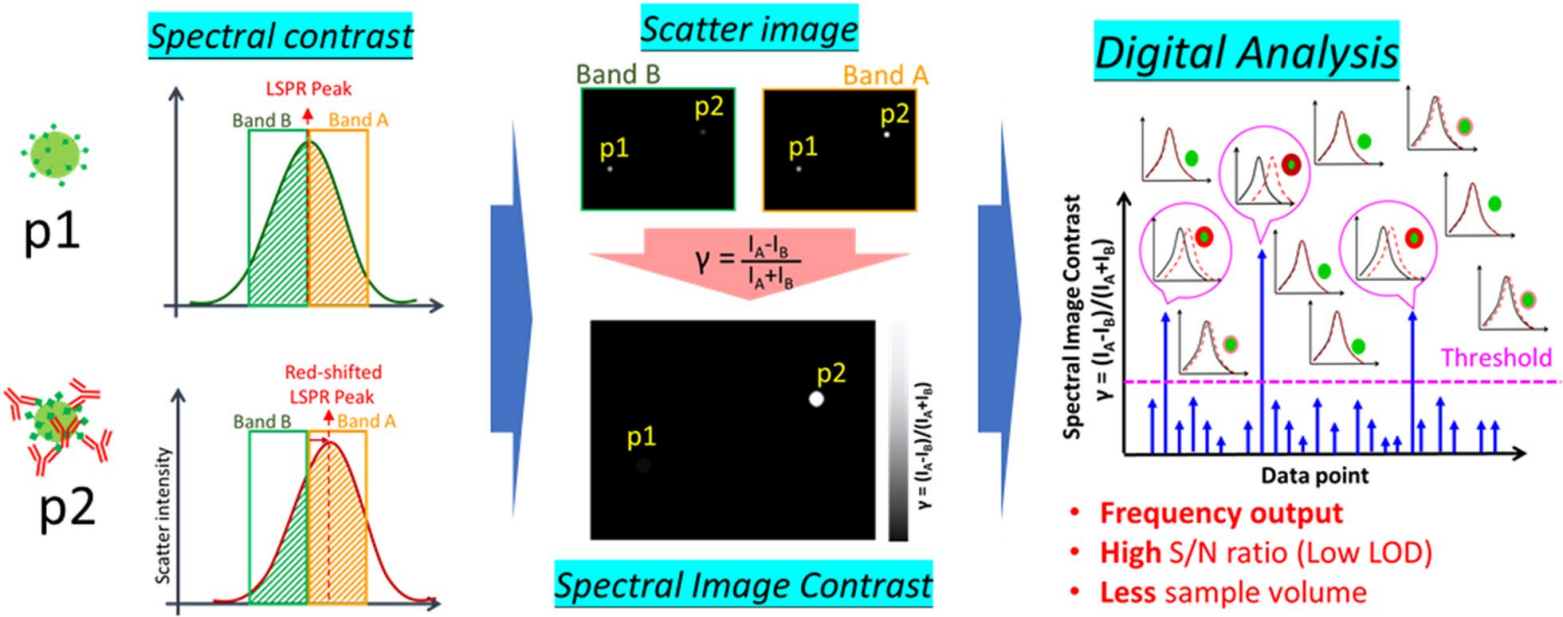

**Supplementary Information:**

The online version contains supplementary material available at 10.1186/s12951-021-01188-6.

## Background

Local surface plasmon resonance (LSPR) is a particular SPR triggered by electromagnetic illumination. A coherent oscillation of free electrons occurs on the surface of plasmonic nanoparticles (NPs), such as gold and silver nanoparticles (Au, Ag NPs) [1, 2]. Given that the LSPR of Au and Ag NPs shows a resonance peak in the visible wavelength regime, noticeable light scattering or absorption is present [3]. Additionally, the peak resonance wavelength is related to the surface refractive index of plasmonic NPs. This indicates that once analytes attach to the surface, the LSPR peak shifts (typically a redshift). Thus, it can be used as a label-free ruler to quantify analytes. Plasmonic NPs have been widely used for biomolecular sensing, such as for antigens and antibodies, due to these unique properties. They are also well known as LSPR immunoassays for the detection of various diseases [4–7].

There are different LSPR immunoassays that are primarily based on the two conventional methods, UV–visible absorption spectrum (UV–Vis) detection [8–10], and colorimetric detection, including naked-eyed and lateral flow assays (LFAs) [11–14]. UV–Vis methods employ absorption spectra to evaluate biomolecule binding according to the LSPR peak shift of monodispersed NPs. However, the shift is tiny, and the limit of detection (LOD) is restricted mainly by the low figure of merit (FoM) of the LSPR sensor [15]. On the other hand, the naked eye and LFA methods detect analytes based on the intense color change induced by the aggregation of NPs either in the liquid phase or on test papers. Its simplicity, high user-friendliness, and low cost make it the most prevalent assay for point-of-care tests. However, at the same time, significant color changes require of a high number of NPs. Considering the number of analytes loaded on each NP, UV–Vis results in an inferior LOD (ng to μg mL^−1^) if there is no other signal or analyte amplification. Improvements in sensitivity has been slow for a long time, and pushing the boundary of the current LOD is a major challenge of current LSPR immunoassays in biosensing [16].

**Fig. 1:**
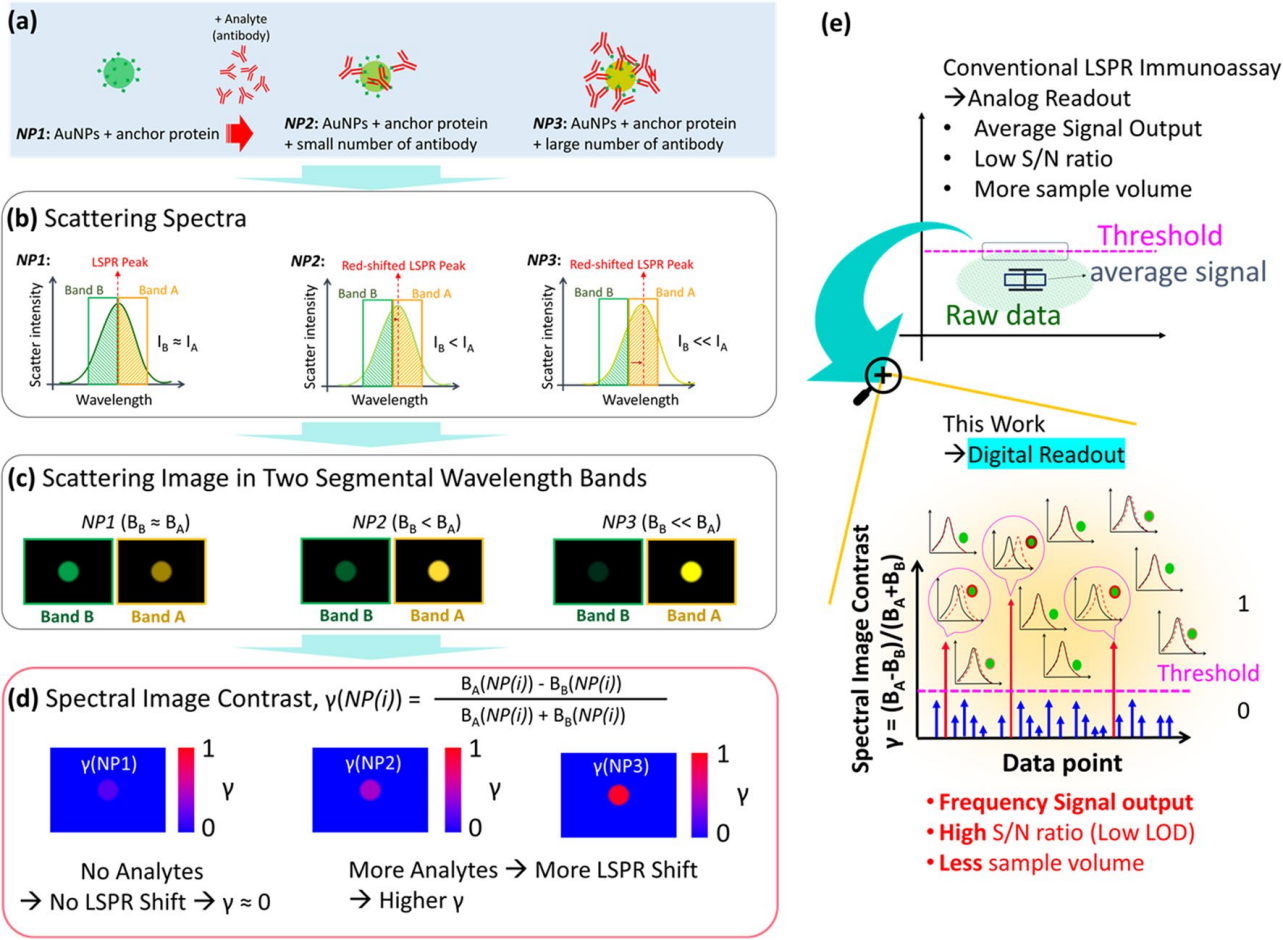
**a** Illustrations of the anchor-protein-modified AuNPs before and after analyte (anti-body) binding. Schematic diagrams of **b** the scattering spectra and **c** the scattering images in response to AuNPs in (**a**). **d** Spectral image contrasts of different AuNPs according to the scatter brightness in (**c**). **d** Schematics of the conventional analog and developed digital LSPR detection. The magenta dashed lines indicate the threshold for the minimum detectable signal of each analysis

To improve sensitivity, we proposed spectral image contrast-based flow digital nanoplasmon-metry (Flow DiNM). The concept of spectral image contrast is based on comparing the brightness in the scattering image of individual AuNPs within two selected wavelength regions [17]. As described before, the biosensing of a AuNP-based system is primarily based on its LPSR peak shift in the spectrum caused by the analyte attaching to the surface and its consequent surface refractive index increasing, as schematically shown in Fig. 1a, b. However, the low FoM of LSPR leads to a very low wavelength sensitivity in biosensing. However, if we set the LSPR peak as the center in the scattering spectra and select two wavelength bands (A and B) adjacent to the center, as shown in Fig. 1b, along with the LSPR shift, the areas (I_A_ and I_B_) in these two bands also changed. It increased in band A and decreased in band B, and the area change was much more intense than the LSPR peak shift. This indicates that using the area change in spectra can amplify the LSPR shift readout to an extent. When the spectra are projected as scattering images, the area is represented by scattering brightness. Figure 1a–c also depicts the brightness change in the scattering images following the analytes attaching to AuNPs and their corresponding spectral changes. At the beginning (*NP1*), the scattered LSPR peak of the AuNPs is close to the border between the two segmental wavelength bands. Its areas in scatter spectra are approximated. When presented as the scatter images filtered by these two selected wavelength bands, it shows a similar brightness. As the analytes are binding (*NP2* and *NP3*), the LSPR peak redshifts and thus presents a much brighter scatter in band A than B, and the brightness is also related to the binding number of the analytes. Based on the brightness change in scattering images, we derived the spectral image contrast

1 $$\upgamma (\text{NP(i)}) = \frac{{B}_{A}(\text{NP(i)}) - {B}_{B}(\text{NP(i)})}{{B}_{A}(\text{NP(i)}) + {B}_{B}(\text{NP(i)})}$$

where B_A_(*NP(i)*) and B_B_(*NP(i)*) are the scattering brightness in bands A and B of the AuNP *NP(i)*, respectively. γ is the spectral contrast signal and *i* is the counting number of the AuNP. Generally, as the LSPR redshifts, the brightness from filter A (B_A_) increases while the brightness from filter B (B_B_) decreases. As a result, the contrast change, γ = (B_A_ − B_B_)/(B_A_ + B_B_) shows a much larger increase than the change of the LSPR peak shift. The concept of the dual-segment sensing approach is widely used for sensitive position detection in the spatial domain, such as the cantilever bending measurement in the atomic force microscopy (AFM). Here, we employ this concept for sensitive measurement of peak wavelength shift in the spectral image domain. The difference, B_A_ − B_B_, indicates the analogous spectral contrast related to the LSPR peak shift, which amplifies the brightness change. Normalization to (B_A_ + B_B_) is employed to eliminate the intensity fluctuation from light scattering. As a result, γ is close to 0 at the beginning (*NP1*) and increases as analytes bind (*NP2* and *NP3*) (Fig. 1d). Overall, the spectral image contrast weights and amplifies the LSPR shift readout and provides a quick spectral evaluation in the LSPR immunoassay of AuNPs. The spectral contrast method can easily be combined with a CCD to quickly evaluate LSPR shifts of individual gold nanoparticles. This method offers outstanding peak wavelength linearity, high wavelength resolution, fast response time, and a simple optical system. This capability also facilitates the examination of flowing AuNPs in a fluidic channel, which provides the potential to simply sample and collect thousands of data points in a short time while requiring quick data analysis.

Moreover, in the traditional LSPR immunoassay, the signal is an averaged output of an assembly of AuNPs. Thus, some detected positive signals could be masked by the average signal, and in this case it is difficult to cross the threshold for a minimum detectable signal (as shown in Fig. 1e). Recently, to improve sensitivity, digital analysis has become a trend in biomolecule detection [18–20]. The idea of digital detection is to individually examine ultrasmall detection units, such as every single AuNP, and the output becomes the frequency of the positive signal detected. Digital detection takes advantage of binary decisions to provide a much higher signal-to-noise (S/N) ratio and lower LOD. However, examination of a massive number of data points is also necessary for digital detection to filter out the low number of positive signals at such a low rate. Using spectral image contrast and microfluidic channels, digital LSPR image (D-LSPRI) analysis of AuNPs can be realized. Furthermore, the one-by-one examination method could also achieve a statistically significant results while only needing a low sample volume.

To verify the capability, we compared the LOD of antibody detection to that of the other two conventional LSPR immunoassay methods—UV–Vis and naked-eyed assays. Within the integration of LSPR immunoassay, D-LSPRI, and microfluidic system, the Flow DiNM has an LOD of 1 pg mL^−1^ for anti-BSA, four to seven orders of magnitude lower than that of UV–Vis and naked-eyed assays. Antibodies against the spike protein of SARS-CoV-2 in artificial saliva supplemented with various human proteins and antibodies were also used to validate the sensitivity and specificity in complicated samples. The result shows an LOD of 10 pg mL^−1^ and a broad six-order-of-magnitude dynamic range. The apparent data discrimination from 0 pg mL^−1^ further demonstrates the outstanding sensitivity of the Flow DiNM in complicated samples. Although the LOD raises in complicated samples, the developed Flow DiNM still presents the same LOD grade (10 pg mL^−1^) as ELISA while being label-free, much more accessible, and having a quicker detection time (< 15 min).

## Results and discussions

### Setup of the flow digital nanoplasmon-metry

**Fig. 2:**
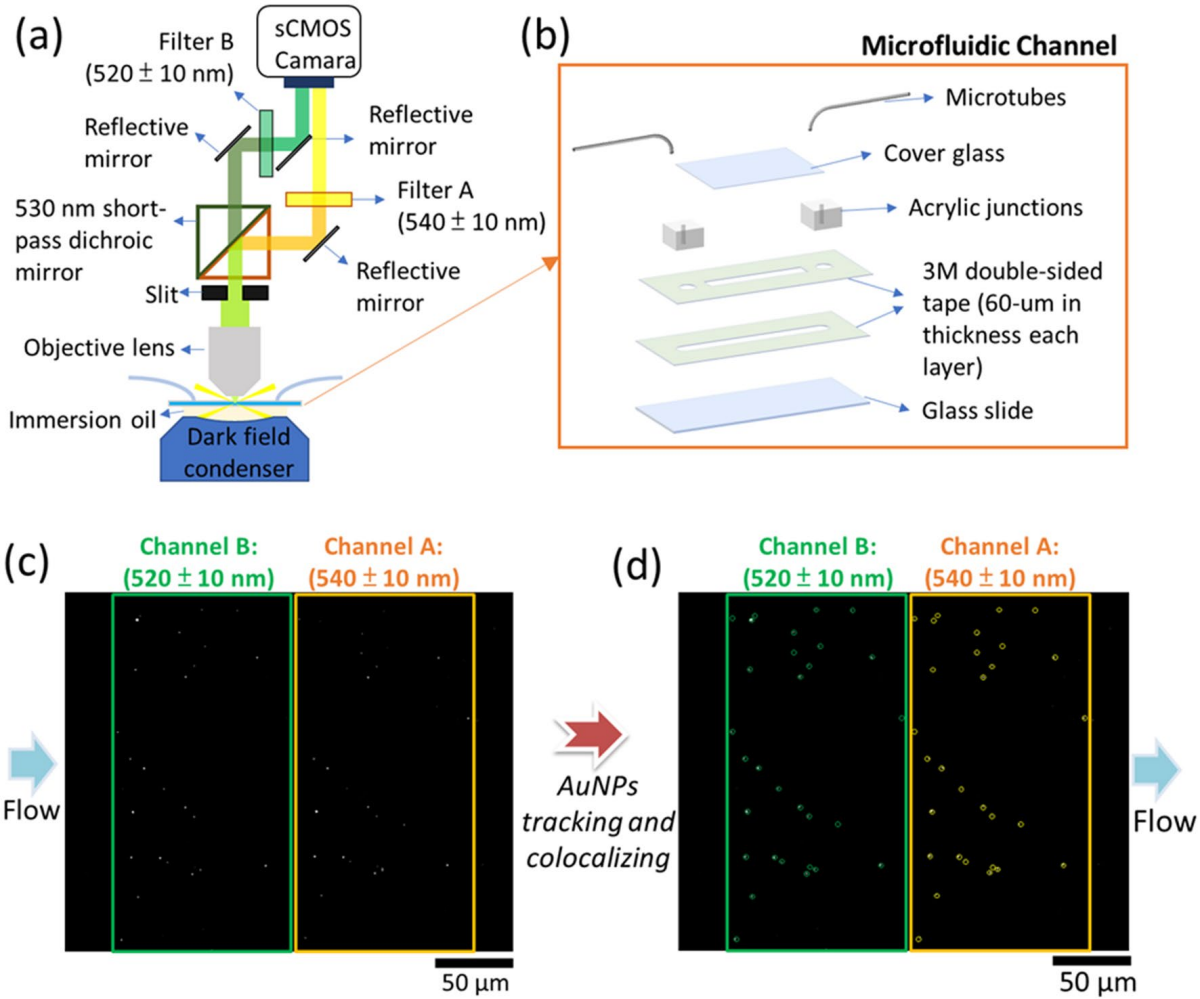
**a** Configuration of the flow digital nanoplasmon-metry (Flow DiNM) based on a darkfield illumination and split imaging system. **b** The nature of the lab-built microfluidic channel. **c** Scatter images of bare AuNPs in split channels A and B and **d** traced by the MATLAB program

Based on the concept of the single AuNP examination described above, flow digital nanoplasmon-metry (Flow DiNM) with an integrated microfluidic chip and darkfield illumination system was developed, as illustrated in Fig. 2a. First, the microfluidic chip leads streamed AuNPs into the dark-field illumination system. It was made of stacking layers of glass slides, acrylic junctions, and double-sided tape. The glass slides were first treated with surface hydrophobic modification by vapor-phase deposition to prevent nonspecific binding from flowing AuNPs. Briefly, glass slides were first put in a chamber together with one mL of trichloro(1H,1H,2H,2H-perfluorooctyl)silane (PTOCTS), and the chamber was then vacuumed sealed and kept at approximately 0.5 atm for 30 min. Glasses were then baked at 120 ℃ for 1 h to facilitate covalent binding between PTOCTS and glass. The contact angle measurement results shown in Additional file 1: Fig. S1 indicate that the glass slides had a hydrophobic surface. Glasses were subsequently sealed and tubed, and the nature of the microchannels was defined by two layers of double-sided tape (3 M, thickness = 60 μm), as shown in Fig. 2b.

The dark-field illumination system can quickly record the LSPR information of each AuNP flowing in the microfluidic chip. It was based on an Olympus upright microscope. A 60× (NA = 0.7) air-type objective and a dark-field condenser (Cyto Viva) with a 20-Watts halide light source were used in this work. AuNPs were drawn into the microchannel described above in the following experiments, and the light scattering of AuNPs was excited by dark-field illumination. The scattering was then passed through a 530-nm shortpass dichroic mirror, in which the cutoff is located at the peak of the LSPR scattering of the bare AuNPs, and separated into two channels with two color bands (green and yellow). Light in each channel was then passed through two bandpass filters to further extract significant bands of LSPR of AuNPs. The two filters used had nonoverlapping neighboring filter bands, and the transmitted wavelength bands were 540 ± 10 nm (filter A) and 520 ± 10 nm (filter B). The LSPR spectra of AuNPs were transferred into intensity information using a dichroic mirror and two bandpass filters. They were quickly recorded as images by a scientific complementary metal-oxide-semiconductor (sCMOS) camera. Figure 2c also shows the actual scattering images of bare AuNPs in split parallel channels A and B, and the bright spots indicate single AuNPs. For data collection, AuNPs were first tracked in channel A (yellow circled) and colocalized in channel B (green circled), as shown in Fig. 2d. Meanwhile, the intensities of AuNPs in the parallel channels were also recorded for the following digital LSPRI analysis.

### Digital-LSPR-image analysis

**Fig. 3:**
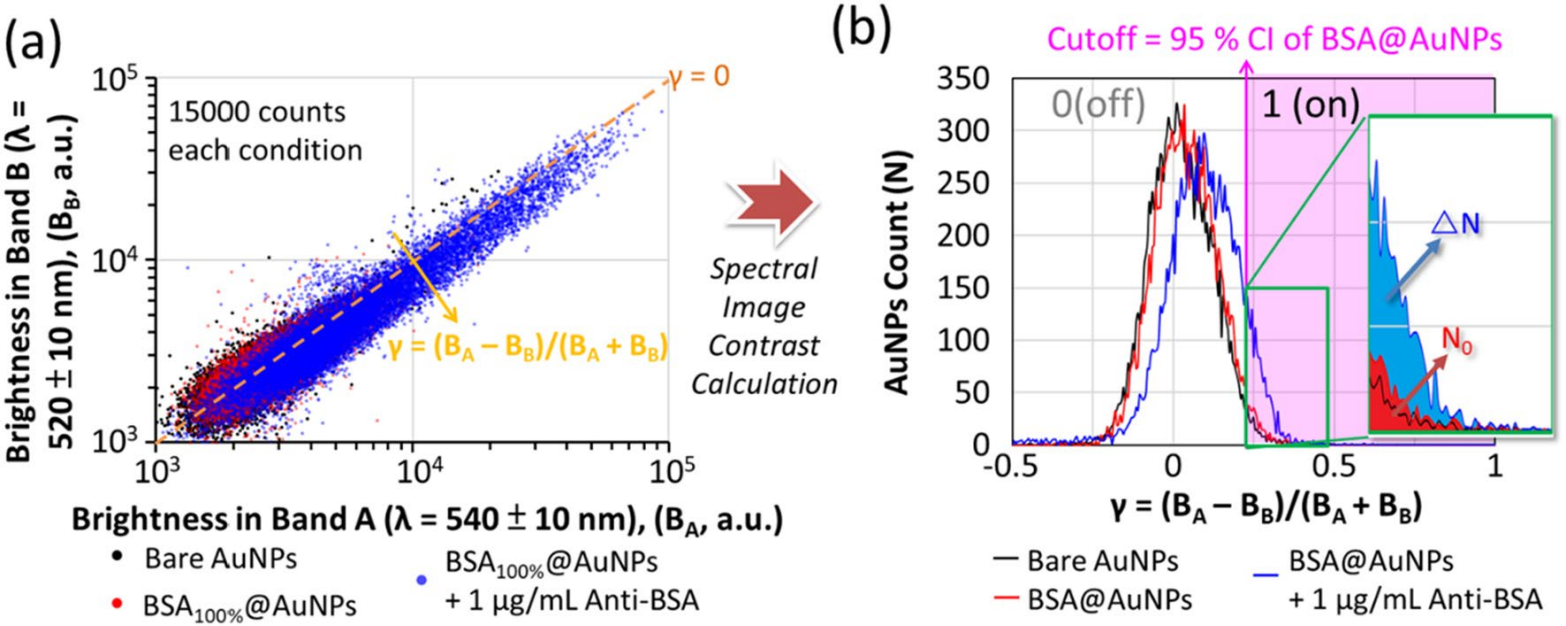
**a** Brightness scatter plot of the bare AuNPs (black dots) and the BSA-modified AuNPs (BSA_100%_@AuNPs) before (red dots) and after (blue dots) anti-BSA conjugation. **b** AuNP counts versus spectral contrast γ = (B_A_ − B_B_)/(B_A_ + B_B_) distribution extracted from (**a**)

Figure 3a shows the intensity scatter plot of the bare AuNPs and the BSA-modified AuNPs (BSA_100%_@AuNPs) before and after anti-BSA conjugation. Each dataset contains 15,000 data points, the detection time was less than 15 min, and there was a linear distribution between the B_A_ and B_B._ There were three component factors, B_A_, B_B_, and the location in the plot. Although the location of the data points is slightly inclined toward weaker B_B_ values and more robust B_A_ values after BSA modification and the conjugation of anti-BSA, it is still too complicated to evaluate the dataset from these coordinates. However, by applying the spectral image contrast, Eq. 1, to the raw data, the dataset can be transferred to linearly uncorrelated variables, which are the counts (N) and γ = (B_A_ − B_B_)/(B_A_ + B_B_) here, as shown in Fig. 3. By redefining a new orthogonal coordinate system, the LSPR shifts of AuNPs are optimally described in a digital dataset. The brightness difference (B_A_ − B_B_)) between the selected wavelength bands indicates the analogous spectral contrast related to the LSPR peak shift [17]. Normalization to (B_A_ + B_B_) efficiently eliminates the intensity fluctuation from light scattering. The counts show the significant LSPR shift statistic distribution and help us study the variation of the dataset trends more straightforwardly.

Figure 3b shows the γ value distributions versus the AuNP counts, which exhibit a Gaussian distribution. We found that with increase in surface-attached biomolecules (bare, BSA-modified, and then anti-BSA-conjugated), the γ values of most data points increased, and thus, the distribution shifted to a higher γ. This result is consistent with the principle of the LSPR immunoassay that protein binding induces a redshift of LSPR in resonant spectra, as depicted in Fig. 1b. However, there is still a slight difference between the conventional and digital LSPR analyses. In the traditional LSPR analysis, the readout is the average shift of the LSPR peak. For the digital LSPR analysis, the readout is the frequency of positive detection, which signifies that more biomolecule binding induces more AuNPs to cross the LSPR threshold. Thus, the threshold set plays a critical role in digital analysis. In this work, we set a cutoff at the upper 95% confidence interval (CI) of the BSA_100%_@AuNPs as the binary threshold to define positive detection. The upper 95% CI connotes that without the analytes (negative control, N.C.), 97.5% of the dataset lies outside the cutoff and presents as negative detection. In contrast, the remaining 2.5% of the dataset is considered the background (N_0_). The readout would be the relative positive-detection count (ΔN/N_0_) increment following the attachment of analytes, as shown in the inset of Fig. 3b.

### Influences of the probe protein to PEGylation ratio on AuNPs for analysis

As described before, the readout in the digital analysis is the positive-detection number of the immuno-agents, the AuNPs. To increase the sensitivity, the positive-detection number of AuNPs within limited analytes needs to be increased as much as possible. Therefore, the probe proteins modified onto the AuNPs play a crucial role in the sensitivity of the digital analysis. Generally, when the probe protein ratio rises, each AuNP could provide more binding sites to the analytes. However, it might result in an uneven division of the limited analytes in the high probe ratio condition. Some AuNPs capture more analytes, while the others can only obtain fewer or none. In the digital LSPR immunoassay, a minimum amount of analyte on the AuNPs is necessary to cross the threshold. An uneven distribution would result in a drop in the positive-detection counts. On the other hand, the very low probe protein ratio would result in insufficient binding sites on the AuNPs. These are barely detectable even if the binding sites are fully occupied with the analytes. There is a trade-off for the probe protein ratio on AuNPs to maximize the counts of digital detection and optimize the sensitivity.

**Fig. 4:**
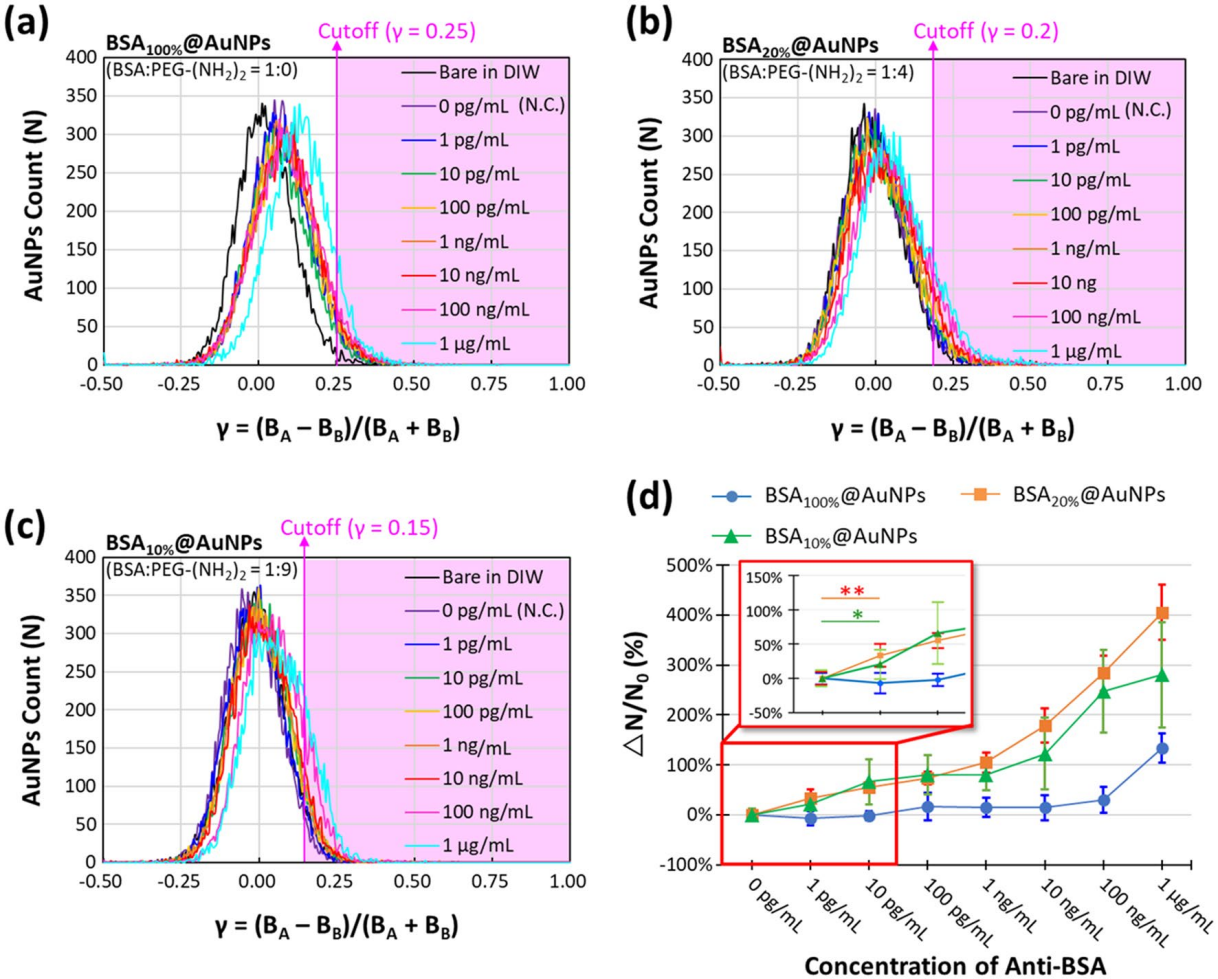
AuNP counts versus spectral contrast γ = (B_A_ − B_B_)/(B_A_ + B_B_) distributions of **a** BSA_100%_@AuNPs, **b** BSA_20%_@AuNPs, and **c** BSA_10%_@AuNPs conjugated with different concentrations of anti-BSA. **d** ΔN/N_0_ signals in response to various concentrations of anti-BSA conjugated to BSA_100%_@AuNPs, BSA_20%_@AuNPs, and BSA_10%_@AuNPs. The sample number of each condition was six, and the error bar is represented by ± SD. Comparisons between 0 and 1 pg mL^−1^ were made using a one-tailed Student’s *t*-test. *p = 0.047 < 0.05; **p = 0.006 < 0.01

In the experiment, we used PEG-(NH_2_)_2_ to adjust the probe protein ratio on AuNPs. PEG-(NH_2_)_2_ is a flexible linear polymer that can be immobilized onto AuNPs via strong physisorption, similar to probe proteins [21, 22]. Meanwhile, the addition of PEG-(NH_2_)_2_ can facilitate the stability of AuNPs in a complicated matrix [23]. Three ratios of BSA to PEG-(NH_2_)_2_, 1:0, 1:4, and 1:9 in wt%, were modified on AuNPs (marked as BSA_100%_@AuNPs, BSA_20%_@AuNPs, and BSA_10%_@AuNPs, respectively), and Additional file 1: Fig. S2 shows their UV–Vis absorption spectra. The BSA/PEG-modified AuNPs interacted with different concentrations of anti-BSA solution. The anti-BSA binding sensitivities and dynamic ranges were compared. Additional file 1: Fig. S3 shows the B_A_ versus B_B_ scatter plots, and Fig. 4a–c shows their corresponding γ distributions before and after anti-BSA conjugation at various concentrations. We find that the γ-distributions vary with the surface modifications on AuNPs. Increasing the ratio of PEG-(NH_2_)_2_ led to a decreasing cutoff (upper 95% CI of N.C.). This is attributed to the addition of PEG-(NH_2_)_2_ reducing the equivalent surface molecule weight and refractive index on AuNPs, which is consistent with the redshift results of the UV–Vis absorption peak. This result demonstrates the reliability of the developed digital method in detecting the LSPR shift with different surface molecule absorptions on AuNPs.

The detection sensitivities were evaluated by detecting different concentrations of anti-BSA from 0 pg mL^−1^ to 1 μg mL^−1^. From the results displayed in Fig. 4d, the use of BSA_100%_@AuNPs does not appear to have evident signal discrimination in detecting anti-BSA below 100 ng mL^−1^. This is because of the uneven division of the limited analytes and consequently reduced positive-detection counts. Once the anti-BSA molecules are present in sufficient concentrations (> 100 ng mL^−1^), most AuNPs can bind sufficient analytes to cross the cutoff and result in a signal jump. For the BSA_10%_@AuNPs, despite an increase in the signal in correlation with the low anti-BSA concentration, the higher concentration gives rise to saturated binding on AuNPs. The saturation limits the detection dynamic range. In comparison, BSA_20%_@AuNPs exhibit excellent sensitivity and a large dynamic range from 1 pg mL^−1^ to 1 μg mL^−1^ (> six orders of magnitude) without saturation. It is noted that the data deviation in the repeated experiments increased with the amount of PEG-(NH_2_)_2_. This result suggests the nonuniformity of the anchor protein distribution on AuNPs. The *t*-test results of BSA_20%_@AuNPs and BSA_10%_@AuNPs show a statistically significant difference between the N.C. (0 pg mL^−1^) and 1 pg mL^−1^. Considering the overall sensitivity, dynamic range, and standard deviation, BSA_20%_@AuNPs were used as digital LSPR immuno-agents.

### Comparison of flow digital nanoplasmon-metry with traditional LSPR immunoassays

After optimization, the flow digital nanoplasmon-metry (Flow DiNM) performance was compared with that of the traditional LSPR immunoassay, UV–Vis absorption spectra method, and naked-eye-based detection. First, it should be noted that with a limited analyte, a higher number of immuno-agents would lead to fewer analytes loading on each agent, reducing the LOD. Therefore, to optimize the LOD of these three methods, the minimal detectable AuNP concentrations of each method were tested, thereby maximizing their analyte-to-immuno-agent ratio. Additional file 1: Fig. S4 displays the results of detecting various concentrations of 50-nm bare AuNPs. The minimal detectable concentration was 5 × 10^9^ NP mL^−1^ for the naked eye and 5 × 10^8^ NP mL^−1^ for the UV–Vis absorption spectra and Flow DiNM.

**Fig. 5:**
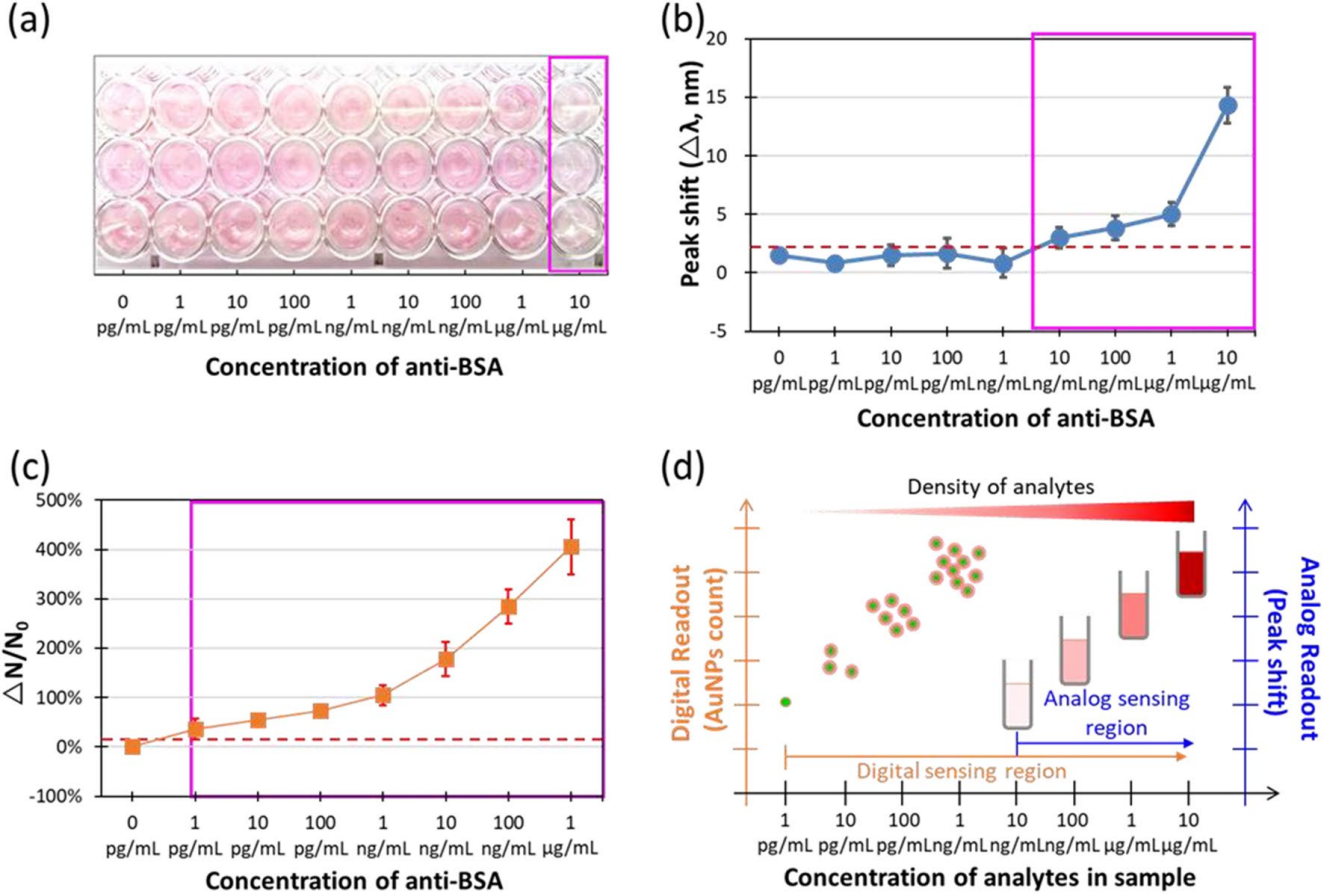
Readout signals of **a** naked-eye, **b** UV–Vis absorption spectra, and **c** Flow DiNM in response to various concentrations of anti-BSA conjugated to BSA_20%_@AuNPs. The red lines indicate the readout signal of LOD, and the magenta rectangles indicate the detecting dynamic range in this pilot experiment. The sample number of each method was six, and ± SD represents the error bar. **d** The schematic plot summarizes the LOD and dynamic range that the analog (UV–Vis absorption) and digital (Flow DiNM)-based LSPR immunoassay can achieve in this work. The format of this schematic plot was based on reference [20]

In this comparison, BSA_20%_@AuNPs with the minimal detectable concentration were used as the immuno-agent for each method to detect various concentrations of anti-BSA. The corresponding UV–vis absorption spectra, hydrodynamic diameters (*D*_*h*_), and zeta potentials (*V*_*ζ*_) are shown in Additional file 1: Figs. S5 and S6. As BSA was modified and the concentration of anti-BSA increased, the LSPR peak shifted, *D*_*h*_ increased, and *V*_*ζ*_ decreased. This indicates that the anchor protein and antibody were functional. Figure 5 shows the compared results of the naked eye, UV–Vis absorption and Flow DiNM methods at 10 μg mL^−1^, 10 ng mL^−1^, and 1 pg mL^−1^, respectively. Unlike UV–Vis absorption spectra and Flow DiNM methods, the signal from naked-eye detection is mainly the produced by the aggregation of AuNPs and its consequent color change [24, 25]. Numerous analytes are generally needed to reduce the stability and monodispersity of colloidal AuNPs. In addition, the demand for higher agent concentrations also reduces the analyte-to-agent ratio, which further impairs detection. Eventually, the process would need a much higher concentration of analytes to trigger a visible signal output and result in a much higher LOD. For both the UV–Vis absorption spectra and Flow DiNM methods, the basis of the signal output is from the redshift of the LSPR peak. However, it is intriguing that even when using the same concentration of immuno-agent, the LOD of UV–Vis absorption spectra method is four orders of magnitude higher than that of Flow DiNM. We attributed it to the much lower signal-to-noise ratio within the analog measurement. The signal of the UV–Vis absorption spectra is the average LSPR peak shift, which is an analog output that requires an internally even signal increase for every AuNP. Therefore, some small amount of positive-detection signals might be masked by the average. In contrast, the Flow DiNM provides digital detection that allows binary decisions for each AuNP. Discrete counting enables the detection method to pick out the individual positive detections, thereby increasing the signal-to-noise ratio even under the same analyte-to-agent ratio with analog measurement. Figure 5d shows the LODs and dynamic ranges that the analog and digital LSPR immunoassay can achieve. These data thoroughly demonstrate that digital detection benefits biomolecule detection with orders of magnitude lower LODs and broader dynamic detection ranges, consistent with previous studies [19, 20].

### Detection of the antibody against SARS-CoV-2 spike protein

COVID-19, a highly infectious respiratory disease caused by a newly discovered virus, SARS-CoV-2, has caused millions of deaths worldwide. However, with the development of the vaccine, herd immunity has become the key to restarting social and economic activity. To facilitate the restart, governments are considering an immune passport, which provides a certificate that people have the anti-body to protect them against the virus and not contagious [26–28]. Thus, antibody detection against SARS-CoV-2 plays a crucial role in the coming post-COVID-19 era. Currently, enzyme-linked immunosorbent assay (ELISA) is considered the gold standard in clinical antibody detection because of its high sensitivity (~ 10 pg mL^−1^). However, the compromise between the limit of detection (LOD) and detection time (hours and even days) is always an issue that has hampered testing for years, in addition, this method requires expensive instruments and specialized operators. These drawbacks have pushed scientists to look for more convenient methods while maintaining a similar or higher LOD grade.

**Fig. 6:**
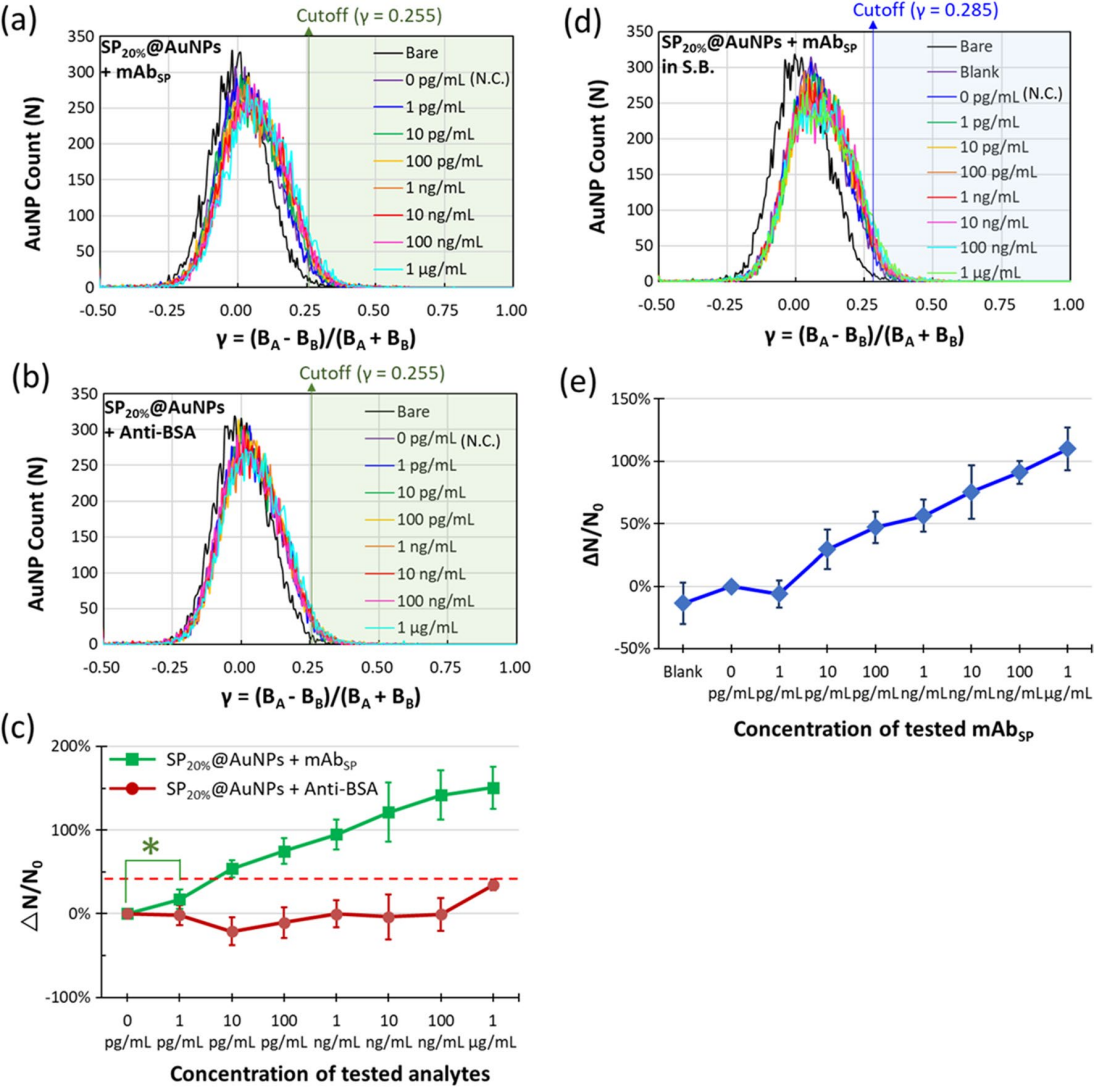
AuNP counts versus spectral contrast γ distributions of SP_20%_@AuNPs conjugated with different concentrations of **a** mAb_SP_ and **b** anti-BSA in TE buffer. **c** Digital readout signals ΔN/N0 in response to mAb_SP_ and anti-BSA. The red lines indicate the readout signal of LOD. **d** AuNP counts versus spectral contrast γ distributions of SP_20%_@AuNPs conjugated with different concentrations of mAb_SP_ in artificial saliva (without any additive proteins, blank) and saliva buffer (S.B.) that contains HSA, IgA, IgG, and IgM. **e** The statistic ΔN/N0 in response to the mAb_SP_. The sample of each condition was six, and the error bar is represented by ± SD. *p = 0.015< 0.05

In the pilot experiments above, we demonstrated the Flow DiNM’s reliability, quick response (≦ 15 min), high sensitivity (LOD ~ 1 pg mL^−1^), and broad dynamics (> six orders of magnitude) in anti-BSA detection. Here, we further demonstrate the capability of the developed Flow DiNM in antibody detection against the spike protein of SARS-CoV-2, and the results are shown in Additional file 1: Fig. S7 and Fig. 6. According to the upper 95% CI of the SP per-modified 50-nm AuNPs (SP_20%_@AuNPs), the cutoff was set to 0.255. Compared to the BSA_20%_@AuNPs, the larger cutoff is attributed to the larger molecular weight (~ 101 kDa) of SP, which is consistent with the UV–Vis absorption spectra and hydrodynamic diameter (D_h_), as displayed in Additional file 1: Figs. S8 and S9. Using SP_20%_@AuNPs as the immuno-agent, the shifts to larger γ values in the AuNP count versus γ distribution can be observed as the mAb_SP_ increases (Fig. 6a). This results in an increasing AuNP count number that crosses the set cutoff. In contrast, the detection of anti-BSA, which was used as the specificity test, did not show any noticeable shift at a concentration of 100 ng mL^−1^ or lower. Even though there was a jump at a concentration of 1 μg mL^−1^, as shown in Fig. 6c, the signals of mAb_SP_ at concentrations higher than 10 pg mL^−1^ were still distinguishable. However, this result also suggests that sensitivity might fluctuate due to the matrix influence of the LSPR immunoassay. To clarify the matrix influence, we further used artificial saliva with and without spiked human antibodies to demonstrate detection in complicated samples.

Studies have demonstrated that SARS-CoV-2 enters the human body mainly via the nasopharynx and can stimulate secretory antigen-specific antibody responses [29–31]. Regardless of whether blood or saliva are used, selectivity in complicated biological media largely determines the accessibility of new LSPR immunoassays. Based on the potential for the onsite sampling requirement of more accessibility and safety in the future, we chose artificial saliva containing human serum albumin and multiple antibodies to mimic human saliva as a complicated sample [32, 33]. The detection of increasing concentrations of mAb_SP_ in mimicked human saliva (saliva buffer, S.B.) was then conducted. Figure 6d, e displays the result, where the blank is the artificial saliva without adding any proteins. As a complicated sample, the cutoff set according to the 95% CI of N.C. is larger than that in the pure sample (TE buffer, Fig. 6a, b). Additionally, the difference in ΔN/N_0_ between the blank and N.C. is approximately 1 μg mL^−1^ of anti-BSA in Fig. 6c. This result is attributed to the matrix influence in the complicated sample. The matrix influence affects the LOD of Flow DiNM. However, the approximate ΔN/N_0_ of the different additives (proteins) and base matrices indicate limited impact. From Fig. 6d, e, although the cutoff was raised, there was still an evidently increasing AuNP count that crossed the set cutoff as the mAb_SP_ concentration increased. The apparent count discrimination from N.C. (0 pg mL^−1^) demonstrates the outstanding capability of the Flow DiNM in detecting antibodies against the SARS-CoV-2 spike protein in saliva. It shows an LOD of 10 pg mL^−1^ and a broad six-order-of-magnitude dynamic range at least. We attributed the LOD variation between anti-BSA and mAb_SP_ detection to the specificity of antibodies and the matrix influence. Although the LOD raised in the complicated sample, the developed Flow DiNM shows the same LOD grade (10 pg mL^−1^) as ELISA while being label-free, much more accessible, and having a quicker readout time.

## Conclusion

Testing is considered a key to controlling epidemics, and antibodies are an essential index to evaluate the efficacy of the developed vaccine and herd immunity in an area in the post-COVID-19 era. This will be beneficial not only for the outbreak of COVID-19 but also the other faster, widespread, and more fatal new pandemics in this era. In this work, we developed a flow digital nanoplasmon-metry (Flow DiNM) method and successfully demonstrated its capability in antibody detection against the BSA and SARS-CoV-2 spike proteins in pure and complicated samples. By integrating local surface plasmon resonance (LSPR) immunoassay, digital local surface plasmon resonance imaging (D-LSPRI), and the microfluidic system, Flow DiNM has the advantages of being label-free, a high sensitivity, a quick readout, and a high sampling accessibility. In the comparison between the UV–Vis absorption spectra method and the counts versus the γ distribution of AuNPs modified with various ratios of BSA and PEG-(NH_2_)_2_, Flow DiNM shows high reliability in discriminating the surface RI on AuNPs. Furthermore, in anti-BSA detection, compared to the conventional analog LSPR immunoassay, the UV–Vis absorption spectra and naked-eye methods, the outstanding performance of high sensitivity and broad dynamic range of Flow DiNM and the digital analysis was successfully demonstrated.

Given their simple, accessible, and noninvasive sampling properties, salivary tests are considered an attractive option in this pandemic era. However, the great challenge of salivary antibody tests is the two to three orders of magnitude lower concentration of the analytes than in serum. To overcome this drawback, ultrasensitive and simple detection methods are urgently needed. In the experiments, Flow DiNM showed a much lower LOD in a complicated sample, 10 pg mL^−1^, than most other analog LSPR immunoassays, as listed in Additional file 1: Table S2 [34–46]. The results also present a significant discrimination and dynamic detection range in complicated saliva samples. Taken together with its quick readout time (less than 15 min) and simple operation, the present study demonstrated the capability of Flow DiNM in rapid detection against the spike protein of SARS-CoV-2 in saliva. In addition to the immune certificate, Flow DiNM can also be used to facilitate the evaluation of the efficiency of vaccine processing, which needs simple, accessible, sensitive, and frequent testing. This would be very valuable to both in science and society. Furthermore, one of the major issues of pandemic antibody tests is the false-positive rate, which is strongly related to the specificity of the developed antibody. It also requires massive clinical data collection for standard comparisons, i.e., identifying whether the providers of the real human saliva or serum samples were infected or received vaccines before sampling. In the near future, Flow DiNM will be tested for standard comparisons to further improve its accessibility in clinical application.

## Materials and methods

### Materials

Poly(ethylene glycol) bis(amine) (PEG-(NH_2_)_2_, Mw 2000), tris-EDTA (TE) buffer (pH = 8), trichloro(1H,1H,2H,2H-perfluorooctyl)silane (PTOCTS), artificial saliva (pH = 6.8), human serum albumin (HSA), immunoglobulin A (IgA from human serum, Product No.: I2636, Lot. No.: 091M4758), immunoglobulin G (IgG from human serum, Product No.: I2511, Lot. No.: 081M4859), and immunoglobulin M (IgM from human serum, Product No.: I8260, Lot. No.: 108M4827V) were purchased from Sigma-Aldrich, Taiwan. It should be noted that the sampling dates of human IgA, IgG, and IgM are all prior to 2019. The outbreak of COVID-19, and thus the samples contain no antibody against SARS-CoV-2. Citrate-capped spherical gold nanoparticles (AuNPs) with a diameter of 50 nm were purchased from nanoComposix, U.S. SARS-CoV-2 spike protein (SP, Mw ~ 101.4 kDa) and corresponding antibodies (SARS-CoV-2 Spike Neutralizing Antibody, Mouse monoclonal anti-body IgG1, mAb_SP_) were purchased from Sino Biological Inc., China. Fetal bovine serum (FBS) was purchased from Invitrogen, Taiwan.

In human saliva, there are albumin and immunoglobulins. Thus, to simulate human saliva as closely as possible, according to reference [32, 33], HSA, IgA, IgG, and IgM were added to artificial saliva at final concentrations of 60 μg mL^−1^, 140 μg mL^−1^, 16 μg mL^−1^, and 4.1 μg mL^−1^ as saliva buffer (SB). It was then stored at 4 ℃ for the following experiments.

### Surface modification and characterization of spherical gold nanoparticles for anti-BSA mAb_SP_ detection

One milliliter of gold nanoparticles (AuNPs) with an initial particle concentration of 4 × 10^10^ particles mL^−1^ was first centrifuged at 5000 rpm for 10 min at 4 °C. Then the suspension was removed. The same volume of TE buffer, which contained 20 μg mL^−1^ anchor protein (BSA for anti-BSA detection or SP for mAb_SP_ detection) and 80 μg mL^−1^ PEG-(NH_2_)_2_, was used to redisperse the AuNPs, and the mixture was kept at 4 °C. After 12 h, the unbound protein and PEG-(NH_2_)_2_ were removed by centrifugation (5000 rpm, 10 min at 4 °C). BSA-coated AuNPs and SARS-CoV-2 spike protein-coated AuNPs (BSA_20%_AuNPS and SP_20%_@AuNPs, 1 mL in microtube) were subsequently washed by centrifugation again and redispersed in tris-EDTA buffer. The final concentration of both BSA_20%_@AuNPs and SP_20%_@AuNPs was adjusted to 5 × 10^10^ particles mL^−1^ as the stock solution and stored at 4 ℃ for the following experiments.

The UV–Vis absorption spectra, hydrodynamic diameters (*D*_*h*_), and zeta potentials (*V*_*ζ*_) of bare AuNPs, BSA_20%_@AuNPs, and SP_20%_@AuNPs were then characterized using a UV–Vis absorption spectra scanner (Biotek Synergy 2) and dynamic light scatters (Brookhaven Instruments, 90 plus Particle Size Analyzer). The concentration of nanoparticles was 5 × 10^8^ NP mL^−1^ for these characterizations. The UV–Vis absorption spectra, hydrodynamic diameters, and zeta potentials of the bare AuNPs, BSA-modified AuNPs, and SP_20%_@AuNPs conjugated with various concentrations of target antibodies in TE buffer are shown in Additional file 1: Figs. S5, S6, S9 and S10, respectively.

Both the redshifting LSPR peak and increasing hydrodynamic diameters indicate the binding of the anchor proteins, and the target antibody was increasingly attached to the surface of AuNPs. In addition, BSA, anti-BSA, and mAb_SP_ were negatively charged when immersed in TE buffer (pH 8), as the isoelectric points of BSA, anti-BSA, and mouse IgG1 were 4.7, 4.8–5.2, and 6.4–7.6, respectively [47, 48]. The decreasing surface charge, the anchor protein modification, and the increasing concentration of target antibodies indicate that the proteins on the AuNPs were functional.

### Working process

The Flow DiNM is carried out in a darkfield illumination system and a microfluidic channel. In the experiments, AuNPs modified with probe protein (BSA or SP) were first mixed with the sample (500 μL) at a final concentration of 5 × 10^8^ NP mL^−1^. The mixture was kept at 37 ℃ for 30 min to facilitate the conjugation of the probe protein on AuNPs to the target analytes (antibodies) in the sample. After that, the mixture was drawn into the microfluidic channel by a syringe pump, and the flow rate was kept at 5 μL min^−1^. It should be noted that when the flow rate is too low, the AuNPs might not flow consistently through the microfluidic channel, as indicated by the arrow in Additional file 1: Fig. S10a and Additional file 2: Movie S1. When this occurs in the region of interest of the scattering image, it results in a repeated analysis of the same AuNPs and somehow reduces the number of significant data points. At a higher flow rate, limited by the sensitivity and exposure speed of the CCD used, the scattering image of the flowing AuNPs would show the distortion presented as a rod rather than a spot, as shown in Additional file 1: Figure S10c and Additional file 4: Movie S3. This would greatly influence the measurement of the location of AuNPs and the subsequent spectral image contrast calculation. Overall, the flow rate of 5 μL min^−1^ (Additional file 1: Figure S10b and Additional file 3: Movie S2) was optimal and allows for sampling a large number of data points without any observable distortion. In contrast, both the too-low and too-high flow rates impaired the digital spectral image contrast analysis. The scattering images of the flowing AuNPs were recorded by the optical setup described before at a frame rate of 1 fps. The scattering intensity contrast of each AuNP in imaging channels A and B was used as digital LSPR image analysis for antibody detection. In each sample, 15,000 AuNPs were counted as a dataset, and the detection time was less than 15 min. After detection, the channel was flushed with Aqua regia and deionized water to remove the AuNP residue inside.

## Supplementary Information

**Additional file 1. **Additional Figures S1–S10, Tables S1, S2.

**Additional file 2: Movie S1.** Flowing AuNPs in the Flow DiNM at a low flow rate of 1 μL per minute.

**Additional file 3: Movie S2.** Flowing AuNPs in the Flow DiNM at an optimal flow rate of 5 μL per minute.

**Additional file 4: Movie S3.** Flowing AuNPs in the Flow DiNM at a high flow rate of 20 μL per minute.

## Data Availability

All data generated or analysed during this study are included in this published article and its additional information files.
