# Supplementary material for: Spectral image contrast-based flow digital nanoplasmon-metry for ultrasensitive antibody detection
Source: J Nanobiotechnology. 2022 Jan 4;20:6. doi: 10.1186/s12951-021-01188-6 (PMC8724237; doi:10.1186/s12951-021-01188-6)
Supplement: Supplementary file 1 — Additional file 1. Additional Figures S1–S10, Tables S1, S2. [file 12951_2021_1188_MOESM1_ESM.docx]

Additional Information

Spectral Image Contrast-Based Flow Digital Nanoplasmon-metry for Ultrasensitive Antibody Detection

Sheng-Hann Wang,^a^ Chia-Wen Kuo,^a^ Shu-Cheng Lo,^a, b^ Wing Kiu Yeung,^a^ Ting-Wei Chang,^a^ and Pei-Kuen Wei^a,^*

^a^Research Center for Applied Sciences, Academia Sinica, Taipei 11529, Taiwan.

^b^Institute of Applied Mechanics, National Taiwan University, Taipei 11221, Taiwan.

*Corresponding author: Pei-Kuen Wei,

128 Academia Road, Section 2, Nankang, Taipei 11529, Taiwan.

Tel.: +886 2 2787 3146

Fax.: +886 2 2787 3122

E-mail address: pkwei@sinica.edu.tw


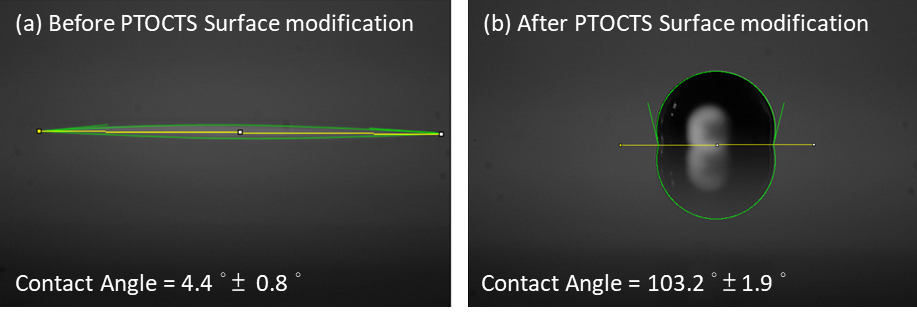


**Figure S1.** Contact angles of the glasses (a) before and (b) after the trichloro(1H,1H,2H,2H-perfluorooctyl)silane (PTOCTS) surface modification. Sample number = 12.


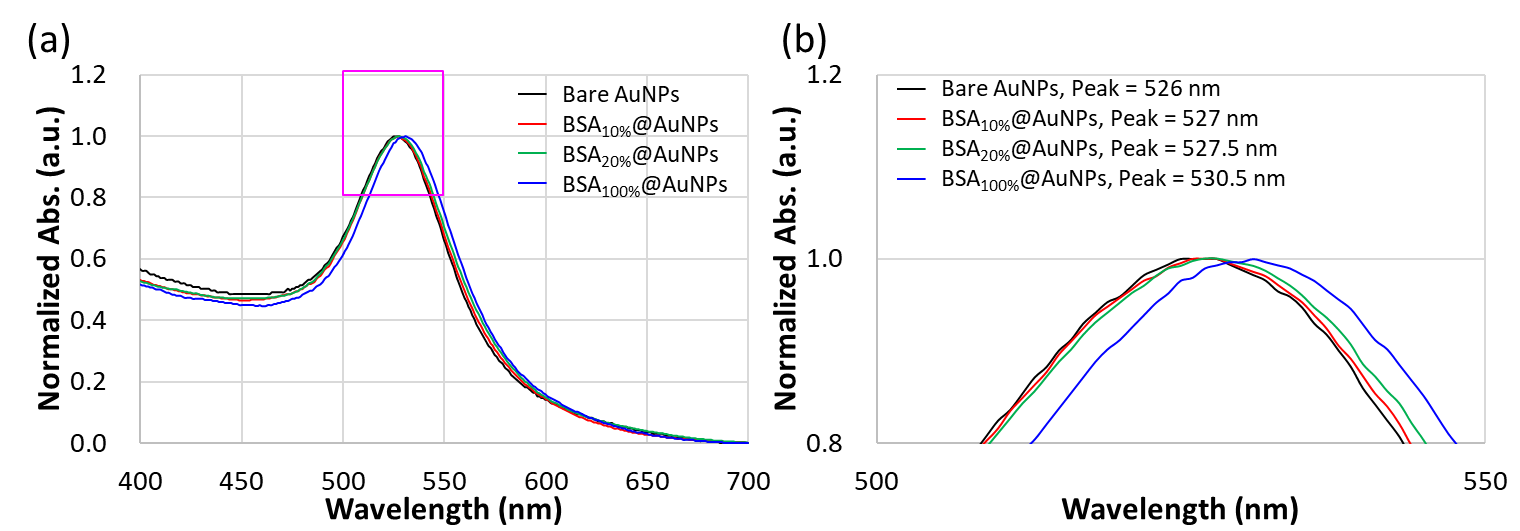


**Figure S2.** UV-Vis absorption spectra of bare and different ratios of BSA to PEG-(NH_2_)_2_ modified AuNPs.


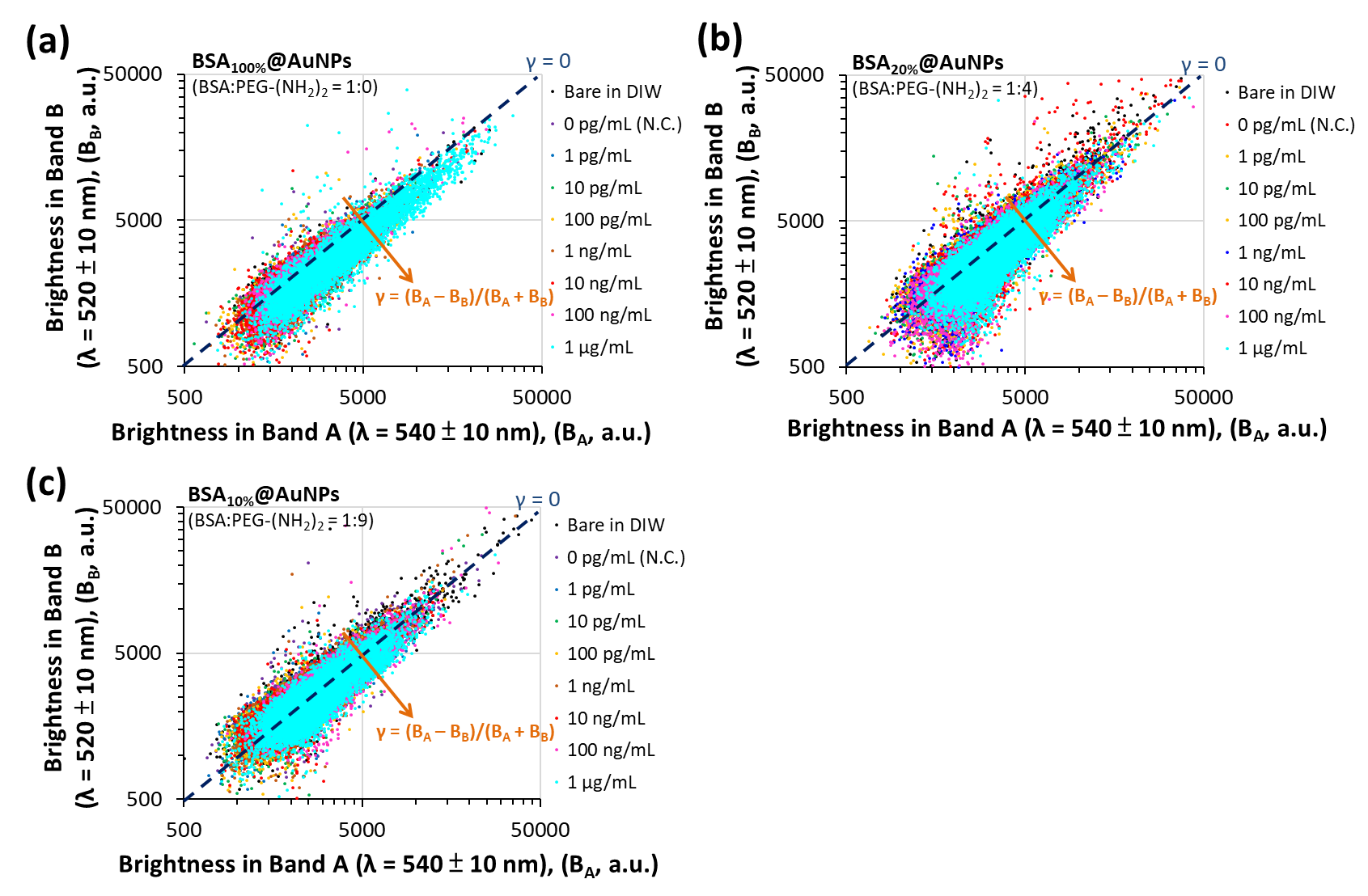


**Figure S3.** Intensity scatters plot of the bare AuNPs, and the different ratios of BSA to PEG-(NH_2_)_2_ modified AuNPs before and after conjugated with various concentrations of anti-BSA. (a) BSA_100%_@AuNPs, (b) BSA_20%_@AuNPs, and (c) BSA_10%_@AuNPs.

**Table S1.** Conversion table of the wt% ratio to molecule ratio of BSA and PEG-(NH_2_)_2_.

| **wt% ratio** | **molecule ratio** |
| --- | --- |
| 1:0 | 1:0 |
| 1:4 | 1:100 |
| 1:9 | 1:225 |
| 1:19 | 1:475 |


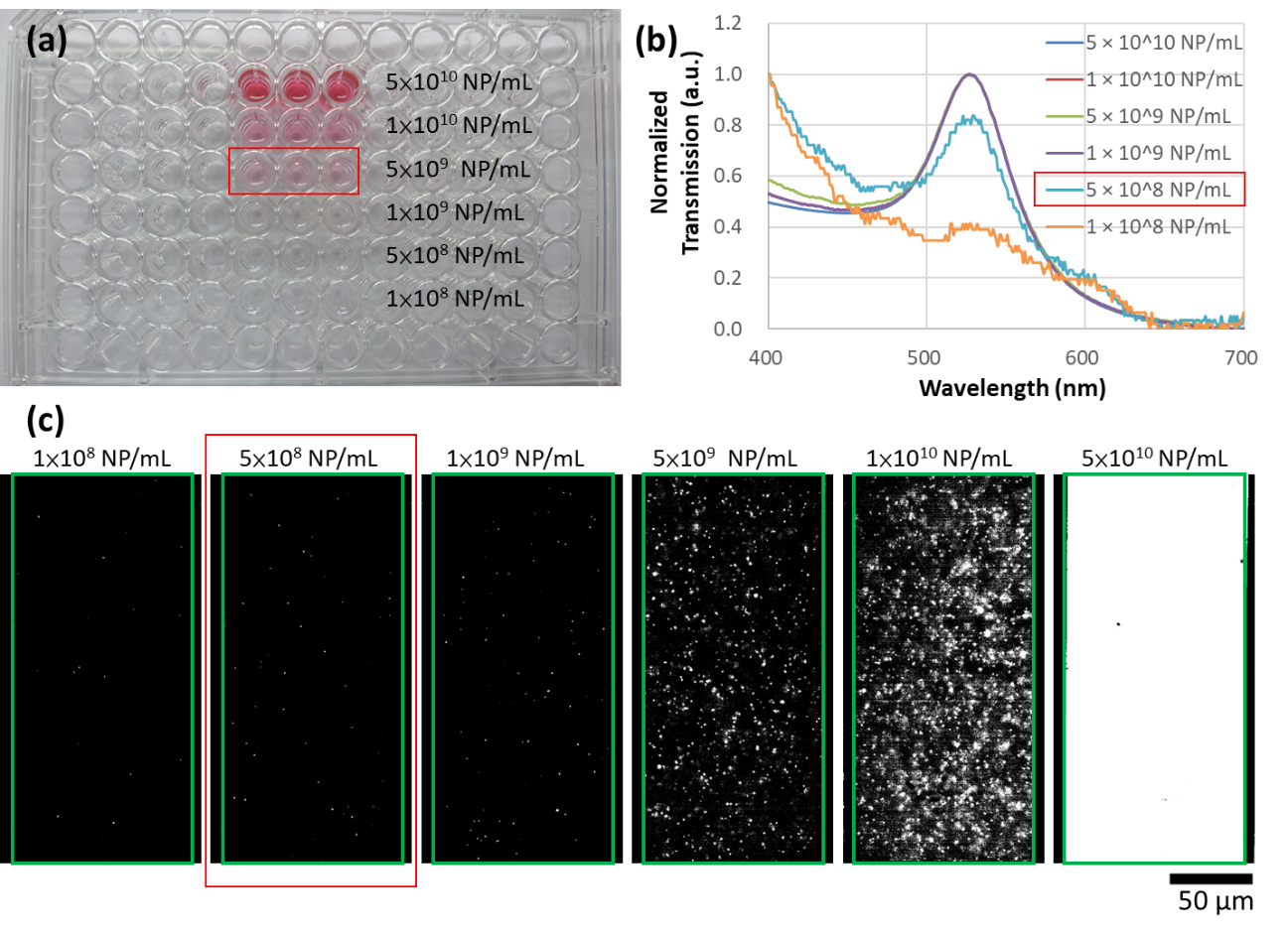


**Figure S4.** Test results of minimal detectable concentration of AuNPs by (a) naked-eye, (b) UV-Vis absorption spectra, and (c) flow digital nanoplasmon-metry (FDNM). The analyte is the 50-nm bare AuNPs with different concentrations. The minimal detectable concentration is 5 × 10^9^ NP/mL for naked-eye and 5 × 10^8^ NP/mL for the UV-Vis absorption spectra and FDNM. Even though the absorption spectrum of the 5 × 10^8^ NP/mL is not as smooth as 1 × 10^9^ NP/mL, the spectrum can still be analyzed and identified by employing the Gaussian fitting.


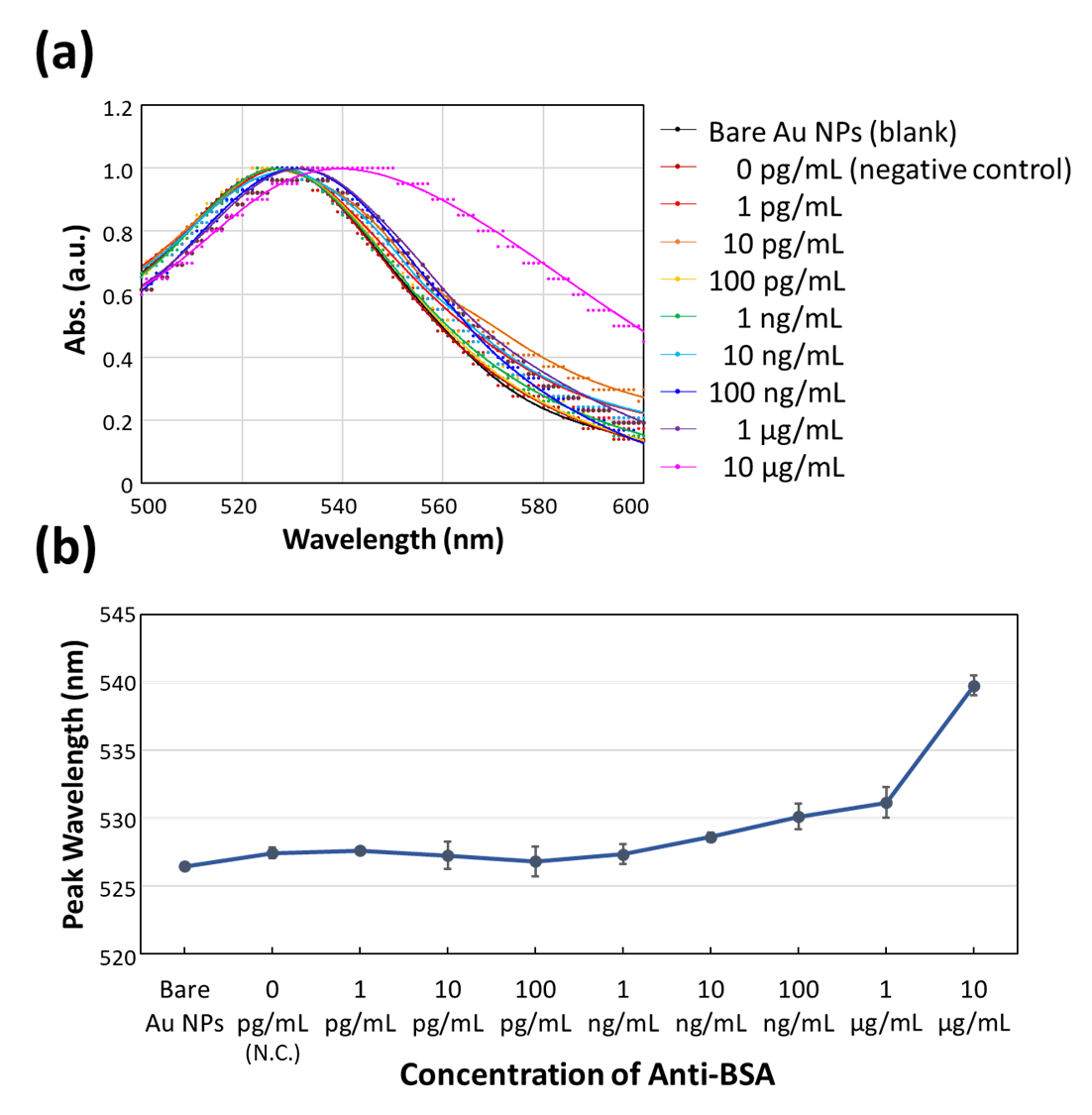


**Figure S5.** (a) UV-Vis absorption spectra of the BSA_20%_@AuNPs conjugated with various concentrations of the anti-BSA in TE buffer and the peaks of LSPR absorption were analyzed and identified by employing the Gaussian fitting as shown in (b).


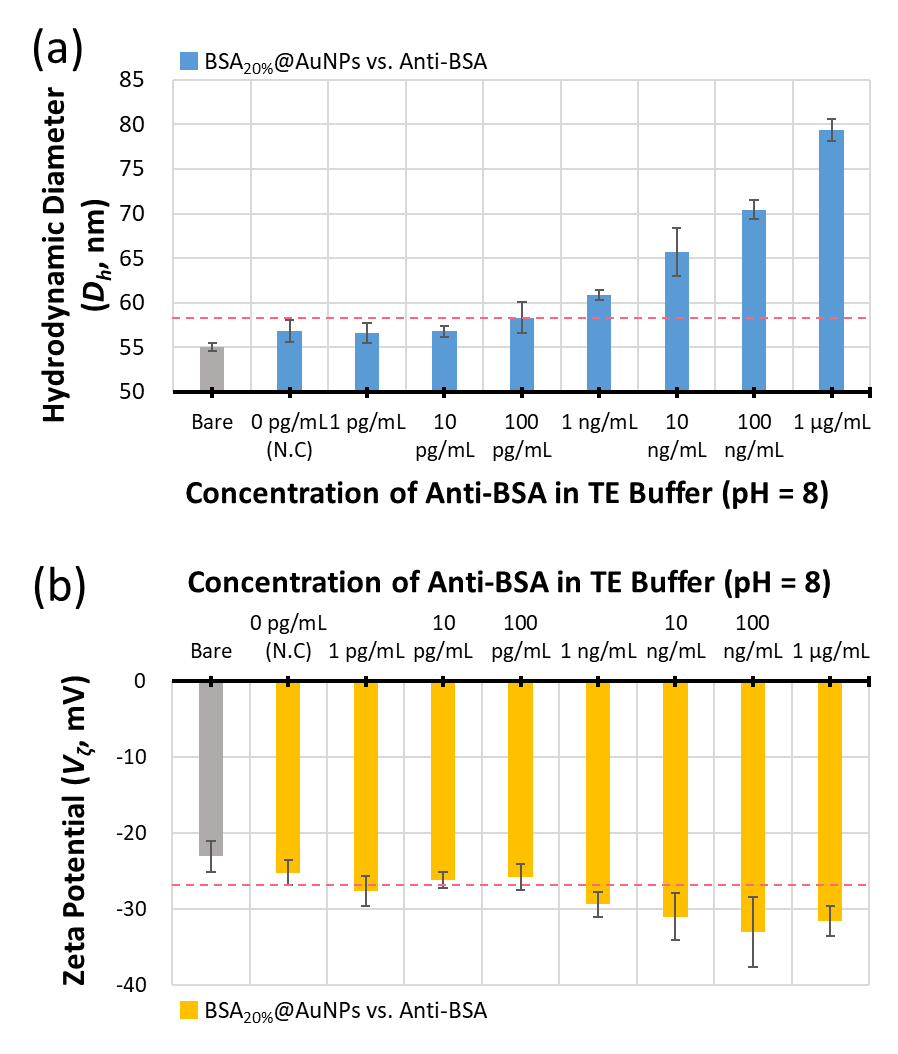


**Figure S6.** (a) Hydrodynamic diameters (*D_h_*) and (b) zeta potentials (*V_ζ_*) of bare AuNPs and BSA_20%_@AuNPs interacted with different concentrations of anti-BSA in TE buffer. Red line indicates the LOD of the ant-BSA detection.


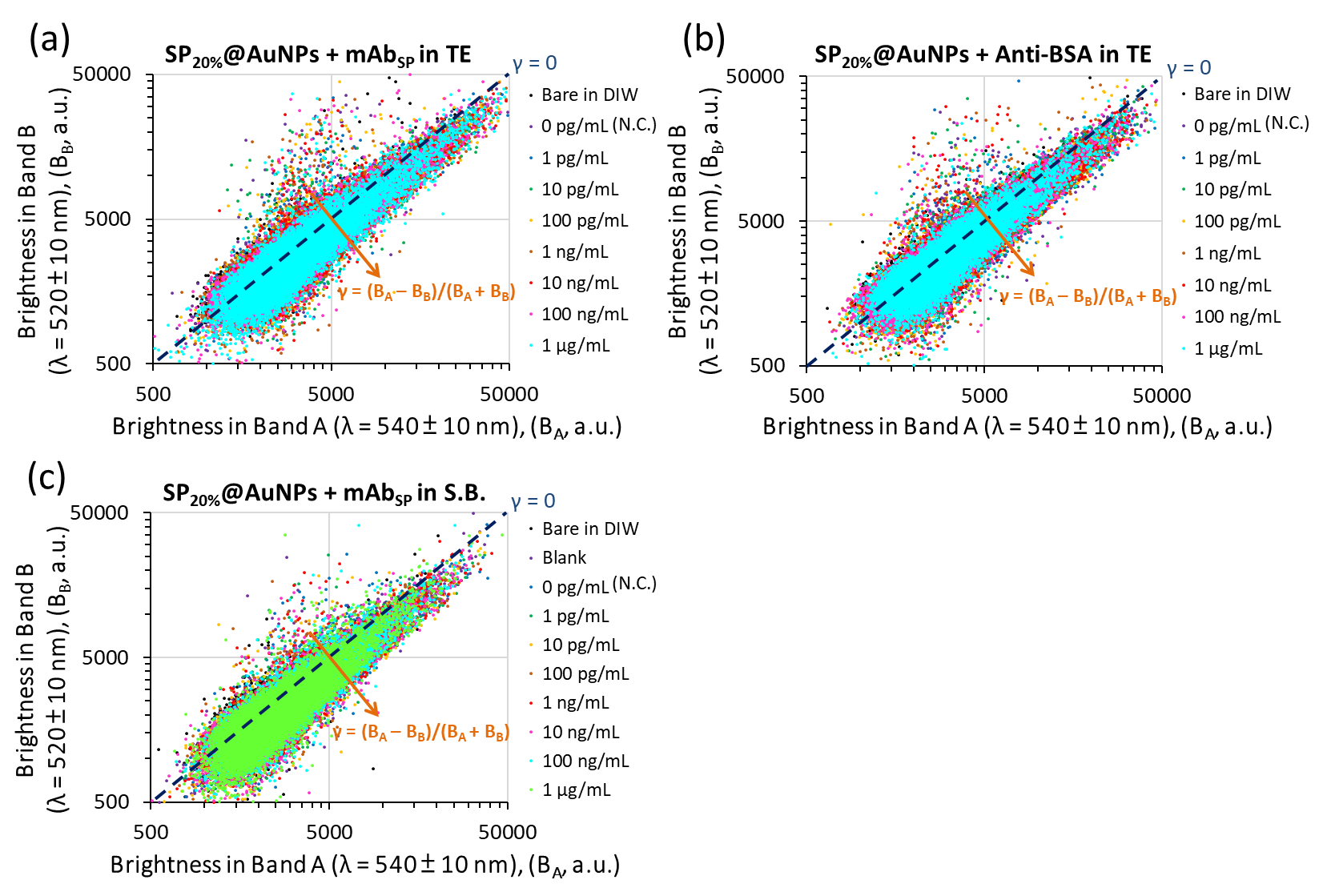


**Figure S7.** Intensity scatters plots of the bare AuNPs and the SP_20%_@AuNPs in response to the various concentrations of (a) the mAb_SP_, and (b) anti-BSA in TE buffer, and (c) mAb_SP_ in artificial saliva (without any additive proteins) and that contains HSA, IgA, IgG, and IgM.


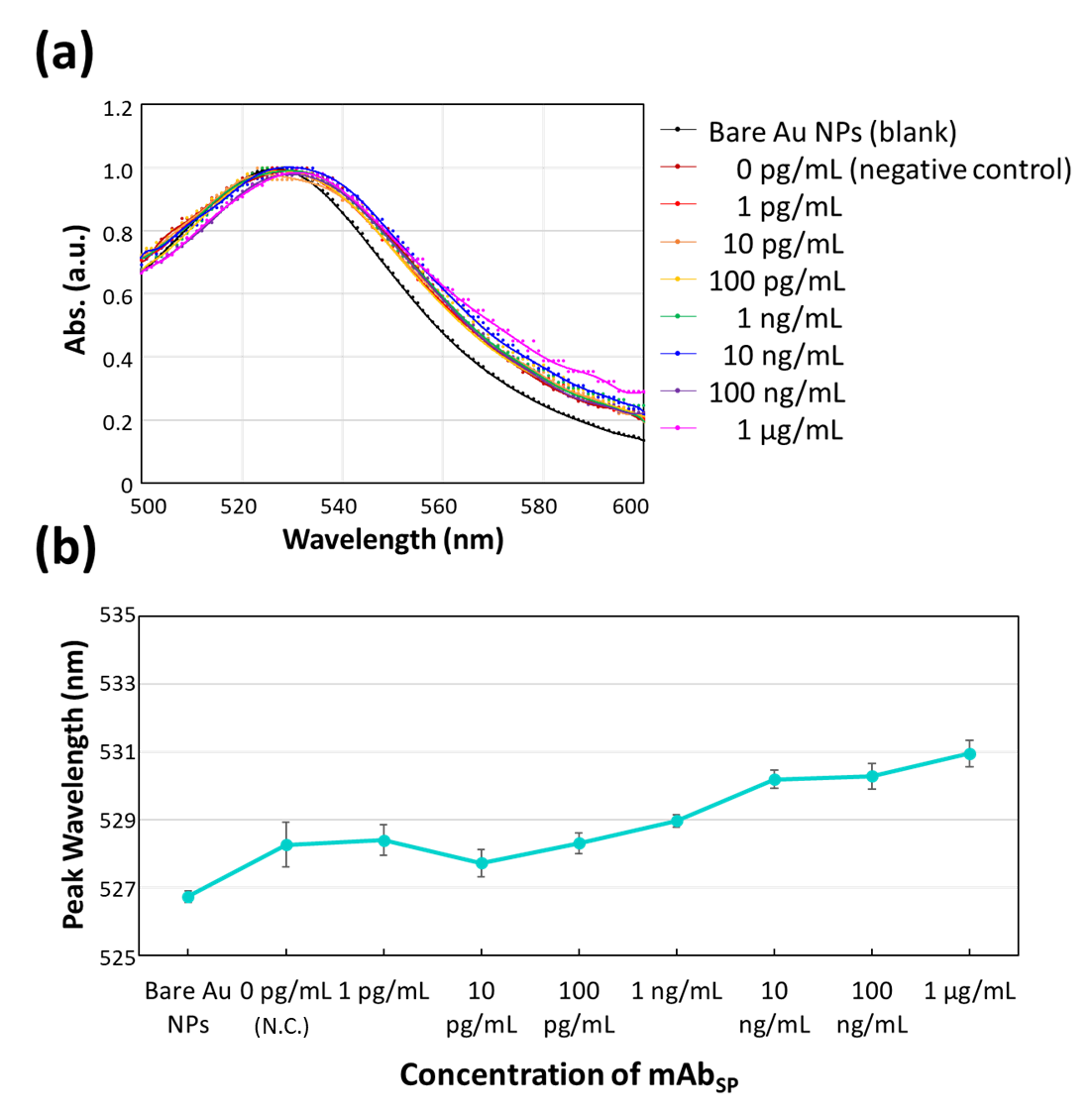


**Figure S8**. (a) UV-Vis absorption spectra of the SP_20%_@AuNPs conjugated with various concentrations of the mAb_SP_ in TE buffer and the peaks of LSPR absorption were analyzed and identified by employing the Gaussian fitting as shown in (b).


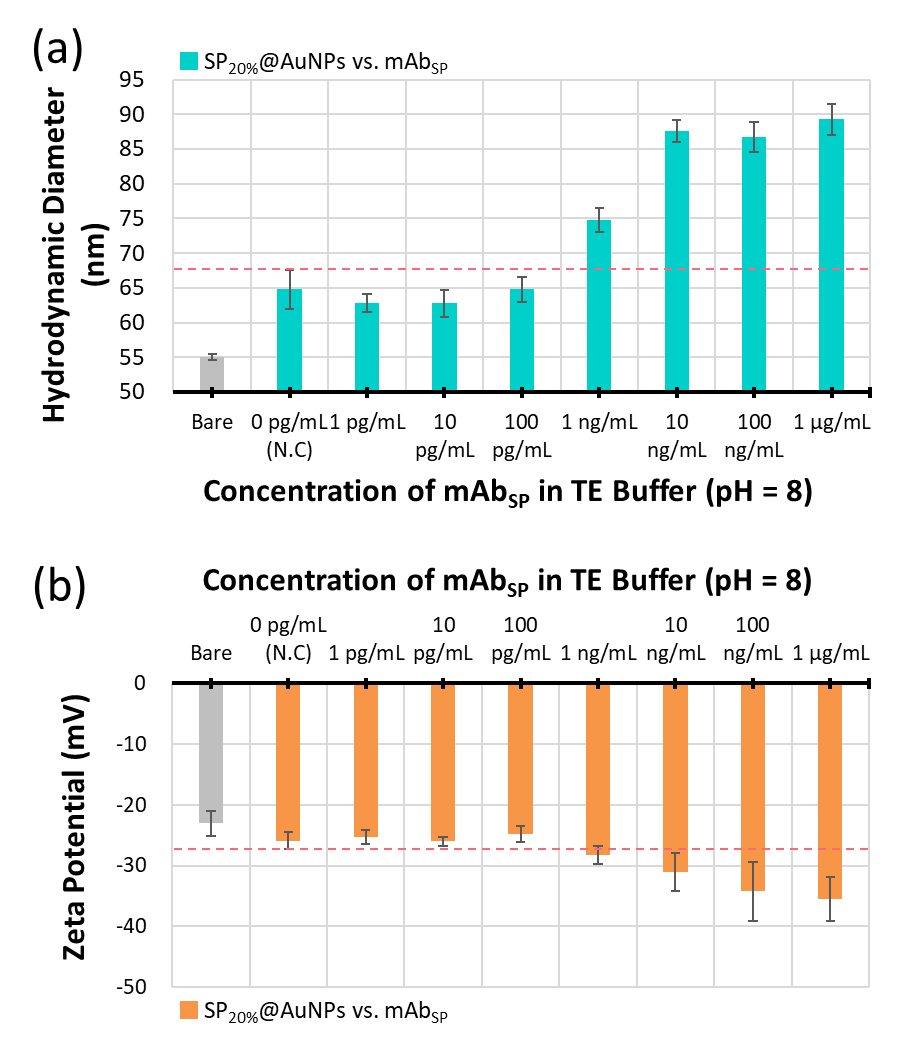


**Figure S9.** (a) Hydrodynamic diameters (*D_h_*) and (b) zeta potentials (*V_ζ_*) of bare AuNPs and SP_20%_@AuNPs interacted with different concentrations of mAb_SP_ in TE buffer. Red line indicates the LOD of the mAb_SP_ detection.

**Table S2.** List of the LSPR-immunoassay in protein detection.

| **Plasmonic structure** | **Analysis** | | **Analytes** | **Signal Amplification** | **Matrix** | **LOD** | **Ref.** |
| --- | --- | --- | --- | --- | --- | --- | --- |
|  | **Detection** | **Analog/Digital** |  |  |  |  |  |
| AuNPs | Absorption Spectra | Analog | Anti-protein A | None | Pure | 1 μg/mL | [1] |
| AuNPs | Absorption Spectra | Analog | Alpha Fetoprotein | None | Complicated | 100 ng/mL | [2] |
| AuNPs | Absorption Spectra | Analog | tumor necrosis factor -alpha (TNF-α) | None | Complicated | 100 ng/mL | [3] |
| AgNPs | Absorption Spectra | Analog | Dengue NS1 | None | Complicated | 60 ng/mL | [4] |
| Au layer on SiO_2_ NPs | Absorption Spectra | Analog | immunoglobulins | None | Complicated | 100 pg/mL | [5] |
| Au Nanospike | Absorption Spectra | Analog | Antibody gainst the SARS-CoV-2 spike protein | None | Complicated | 80 pg/mL | [6] |
| Triangular silver nanosurfaces | Absorption Spectra | Analog | Human Epididymis Protein 4 (HE4) | None | Complicated | 35 pg/mL (1pM) | [7] |
| AuNPs | Colorimetric  (Naked-eyed) | Analog | Thrombin | None | Pure | ~360 ng/mL (10 nM) | [8] |
| AuNPs | Colorimetric  (LFAs) | Analog | Antibodies against the SARS-CoV-2 Virus | None | Complicated | 1 ng/mL | [9] |
| AuNPs | Colorimetric  (LFAs) | Analog | Human IgG | Horseradish peroxidase (HRP) | Pure | 200 pg/mL | [10] |
| AuNPs | Colorimetric  (Fluorescence) | Analog | BSA  β-galactosidase  cytochrome c | Fluorescent dye labeled | Pure | ~5.5μg/mL (110 nM)  ~1.9μg/mL (4 nM)  ~2.6 μg/mL (215 nM) | [11] |
| AuNPs | Colorimetric  (Fluorescence) | Analog | Thrombin | Fluorescent dye labeled | Pure | ¬ 5 ng/mL (0.14 nM) | [12] |
| AuNPs | Spectral Image Contrast | Digital | Anti-BSA  Antibodies against the SARS-CoV-2 SP | None | Complicated | 10 pg/mL | This work |


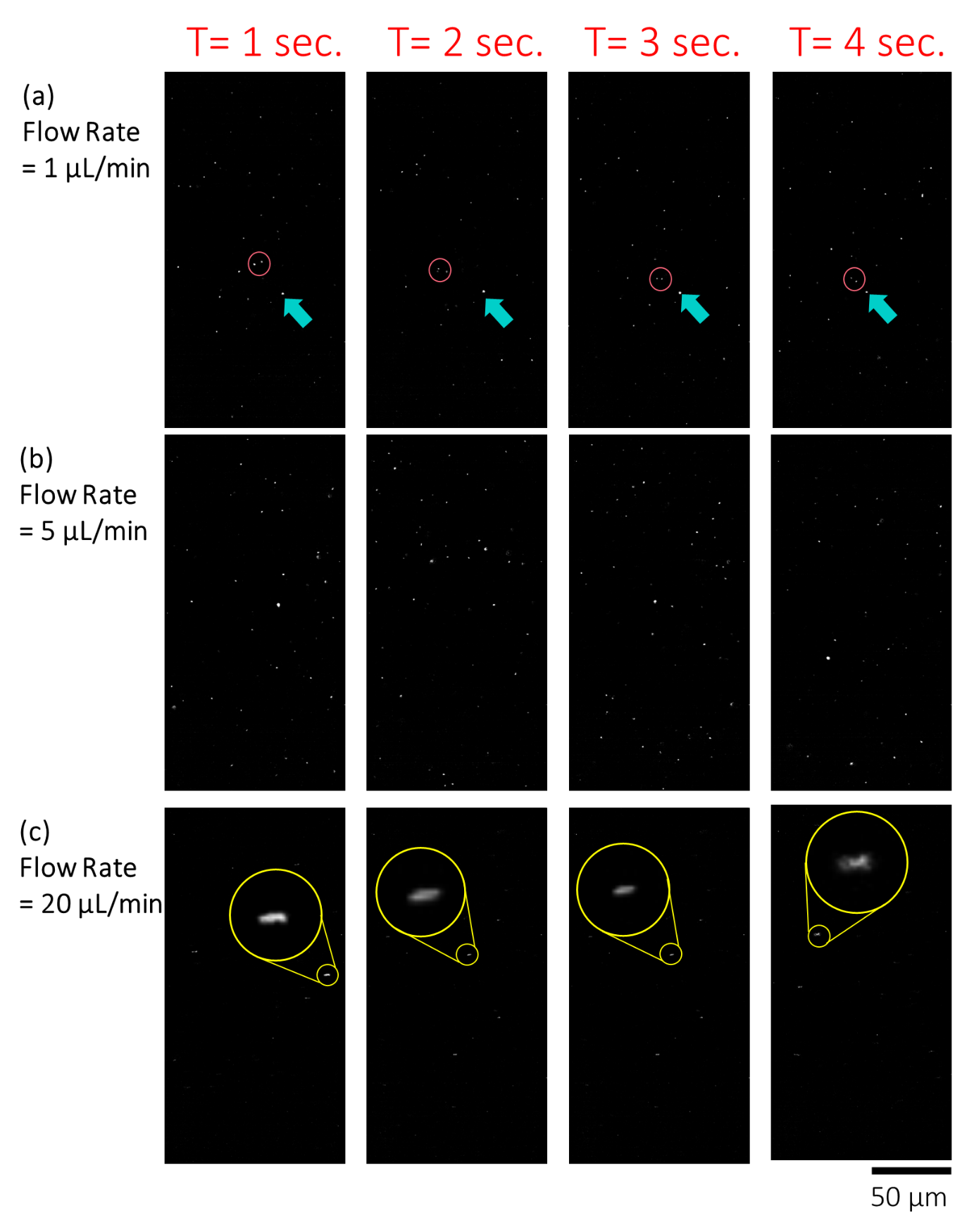


**Figure S10** Scattering images of flowing AuNPs in Band A (540 ± 10 nm) with different flow rate.

**References**

1. Thanh NTK, Rosenzweig Z: **Development of an Aggregation-Based Immunoassay for Anti-Protein A Using Gold Nanoparticles.** *Analytical Chemistry* 2002, **74:**1624-1628.

2. Nietzold C, Lisdat F: **Fast protein detection using absorption properties of gold nanoparticles.** *Analyst* 2012, **137:**2821-2826.

3. Oh B-R, Huang N-T, Chen W, Seo JH, Chen P, Cornell TT, Shanley TP, Fu J, Kurabayashi K: **Integrated Nanoplasmonic Sensing for Cellular Functional Immunoanalysis Using Human Blood.** *ACS Nano* 2014, **8:**2667-2676.

4. Austin Suthanthiraraj PP, Sen AK: **Localized surface plasmon resonance (LSPR) biosensor based on thermally annealed silver nanostructures with on-chip blood-plasma separation for the detection of dengue non-structural protein NS1 antigen.** *Biosensors and Bioelectronics* 2019, **132:**38-46.

5. Endo T, Kerman K, Nagatani N, Hiepa HM, Kim D-K, Yonezawa Y, Nakano K, Tamiya E: **Multiple Label-Free Detection of Antigen−Antibody Reaction Using Localized Surface Plasmon Resonance-Based Core−Shell Structured Nanoparticle Layer Nanochip.** *Analytical Chemistry* 2006, **78:**6465-6475.

6. Funari R, Chu K-Y, Shen AQ: **Detection of antibodies against SARS-CoV-2 spike protein by gold nanospikes in an opto-microfluidic chip.** *Biosensors and Bioelectronics* 2020, **169:**112578.

7. Duan R, Xi M: **A Novel Label-Free Biosensor for Detection of HE4 in Urine Based on Localized Surface Plasmon Resonance and Protein G Directional Fixed.** *Journal of Nanomaterials* 2020, **2020:**8613240.

8. Xia F, Zuo X, Yang R, Xiao Y, Kang D, Vallée-Bélisle A, Gong X, Yuen JD, Hsu BBY, Heeger AJ, Plaxco KW: **Colorimetric detection of DNA, small molecules, proteins, and ions using unmodified gold nanoparticles and conjugated polyelectrolytes.** *Proceedings of the National Academy of Sciences* 2010, **107:**10837-10841.

9. Li Z, Yi Y, Luo X, Xiong N, Liu Y, Li S, Sun R, Wang Y, Hu B, Chen W, et al: **Development and clinical application of a rapid IgM-IgG combined antibody test for SARS-CoV-2 infection diagnosis.** *Journal of Medical Virology* 2020, **92:**1518-1524.

10. Parolo C, de la Escosura-Muñiz A, Merkoçi A: **Enhanced lateral flow immunoassay using gold nanoparticles loaded with enzymes.** *Biosensors and Bioelectronics* 2013, **40:**412-416.

11. You C-C, Miranda OR, Gider B, Ghosh PS, Kim I-B, Erdogan B, Krovi SA, Bunz UHF, Rotello VM: **Detection and identification of proteins using nanoparticle–fluorescent polymer ‘chemical nose’ sensors.** *Nature Nanotechnology* 2007, **2:**318-323.

12. Wang W, Chen C, Qian M, Zhao XS: **Aptamer biosensor for protein detection using gold nanoparticles.** *Analytical Biochemistry* 2008, **373:**213-219.
