# Supplementary figures and images for: Spectral image contrast-based flow digital nanoplasmon-metry for ultrasensitive antibody detection
Source: J Nanobiotechnology. 2022 Jan 4;20:6. doi: 10.1186/s12951-021-01188-6 (PMC8724237; doi:10.1186/s12951-021-01188-6)

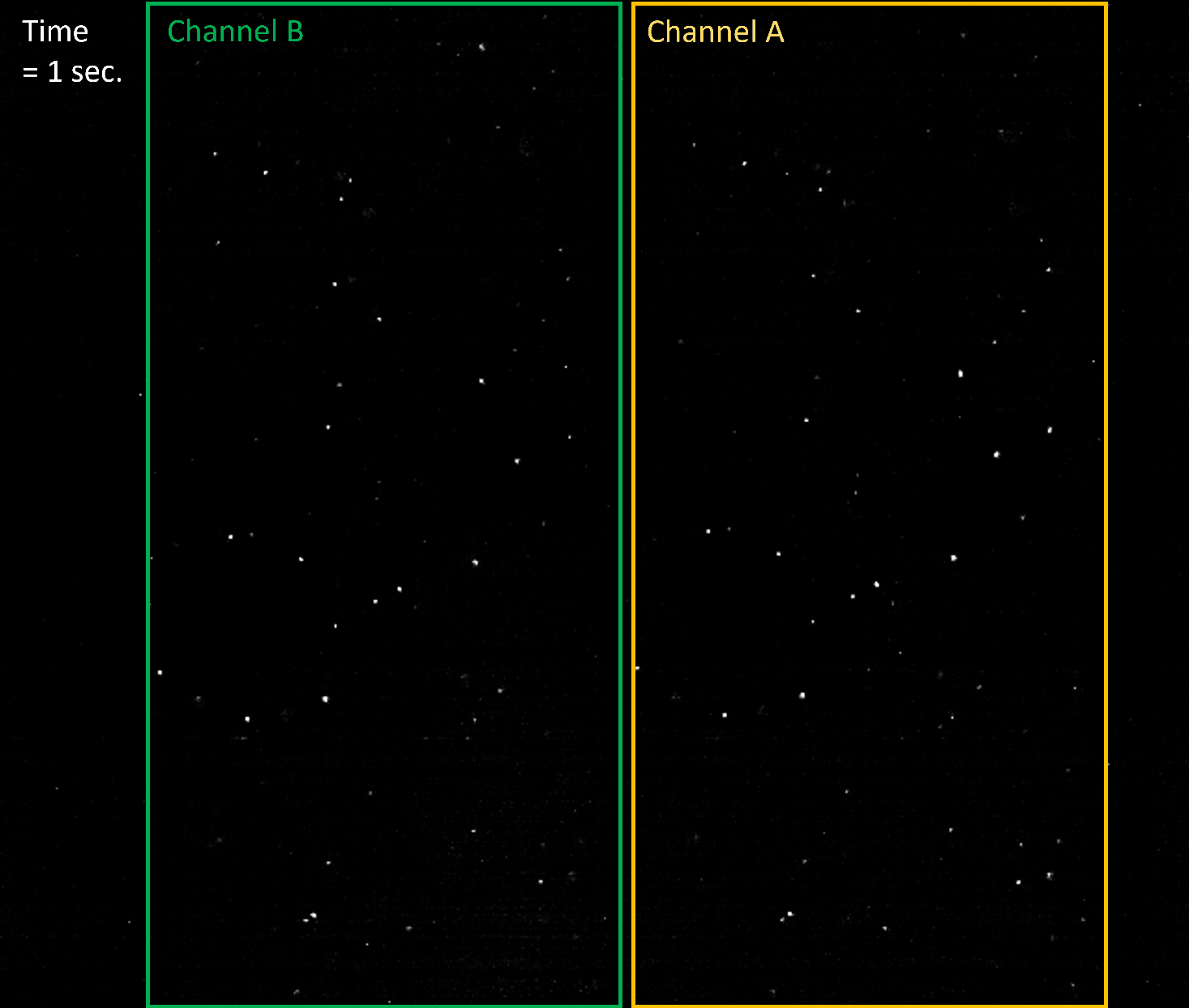

Supplement: Supplementary file 2 — Additional file 2: Movie S1. Flowing AuNPs in the Flow DiNM at a low flow rate of 1 μL per minute. [file 12951_2021_1188_MOESM2_ESM.gif]

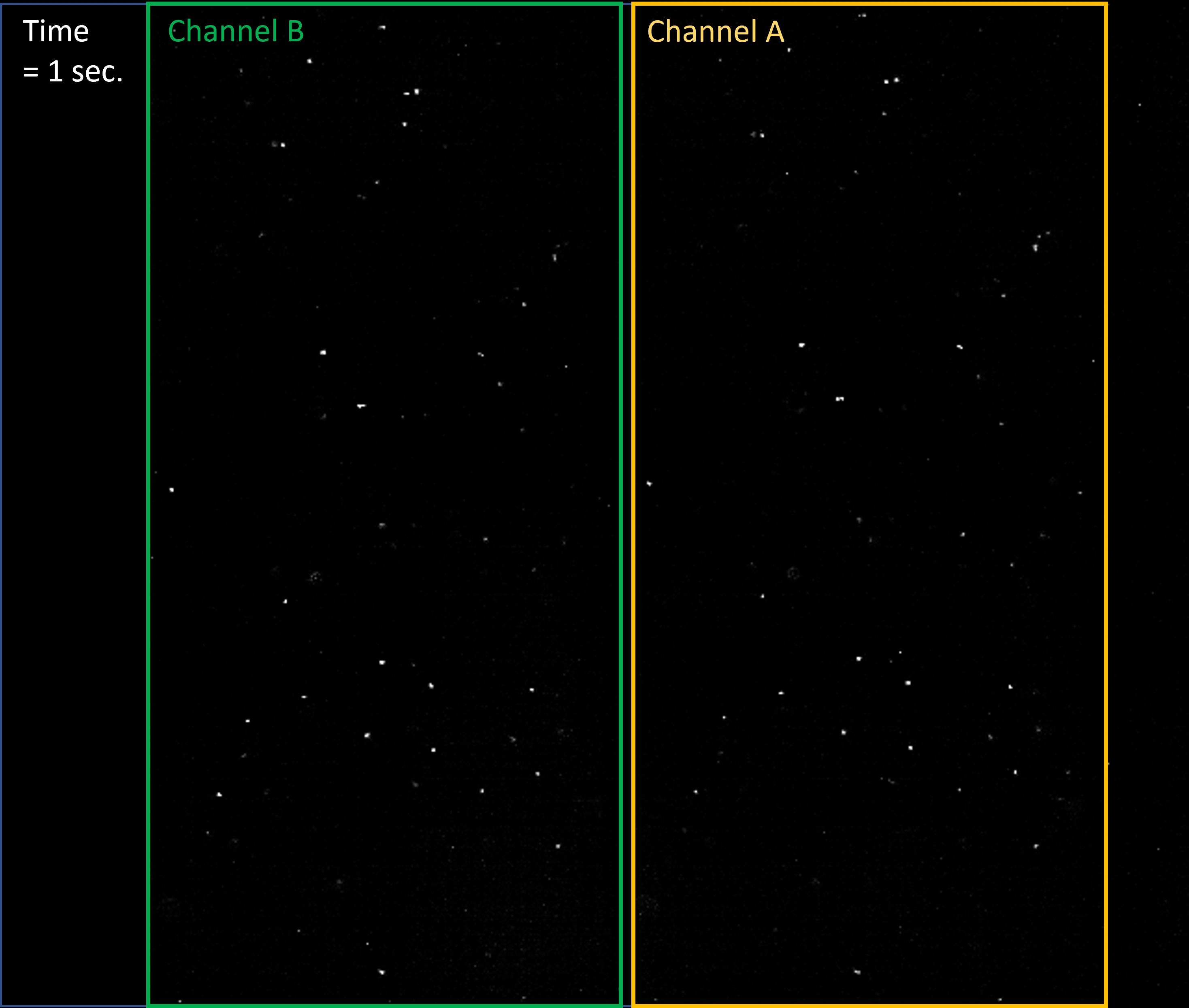

Supplement: Supplementary file 3 — Additional file 3: Movie S2. Flowing AuNPs in the Flow DiNM at an optimal flow rate of 5 μL per minute. [file 12951_2021_1188_MOESM3_ESM.gif]

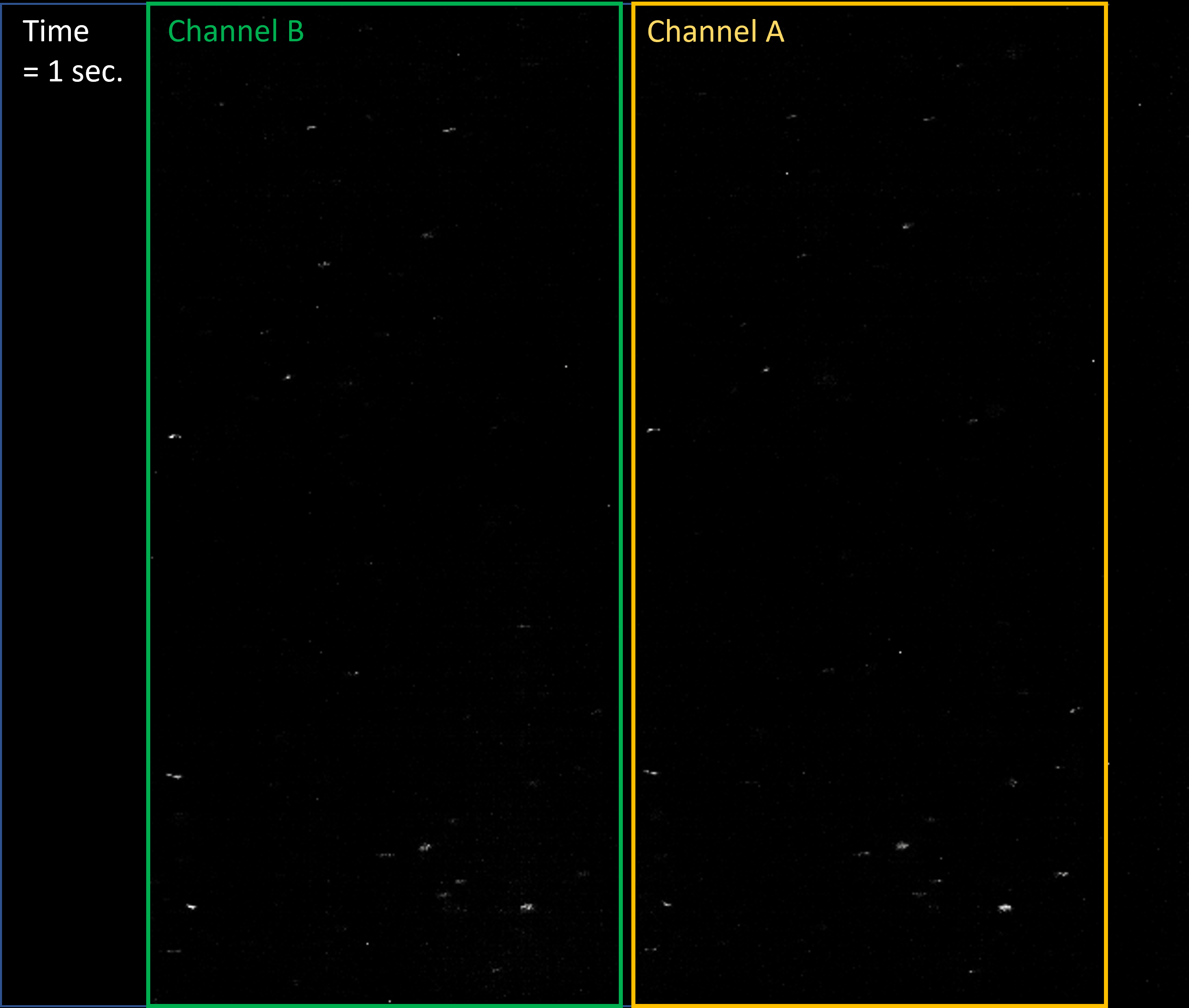

Supplement: Supplementary file 4 — Additional file 4: Movie S3. Flowing AuNPs in the Flow DiNM at a high flow rate of 20 μL per minute. [file 12951_2021_1188_MOESM4_ESM.gif]
